# *Corynebacterium pseudotuberculosis* phospholipase D targets mitochondrial sphingomyelin and induces NLRP3-GSDMD axis-mediated pyroptosis in macrophages to promote infection

**DOI:** 10.1186/s13567-025-01640-7

**Published:** 2025-10-16

**Authors:** Xincan Li, Hong Lv, Chanyu Wu, Hexian Li, Wenyi Yi, Xiaoxin Niu, Qiuyue Peng, Chi Meng, Yongfeng Yuan, Shangquan Tian, Zhiying Wang, Rendong Fang, Zuoyong Zhou

**Affiliations:** 1 College of Veterinary Medicine, Southwest University, No. 160 Xueyuan Road, Rongchang District, Chongqing, 402460 China; 2 State Key Laboratory of Biotherapy and Cancer Center, West China Hospital, Sichuan University, Chengdu, 610041 China; 3 Immunology Research Center, Medical Research Institute, Southwest University, No. 160 Xueyuan Road, Rongchang District, Chongqing, 402460 China

**Keywords:** *C. pseudotuberculosis*, phospholipase D, pyroptosis, macrophages, mitochondria

## Abstract

Infection by *Corynebacterium pseudotuberculosis* (Cp), a facultative intracellular bacterium, causes mainly purulent inflammation and chronic granulomas in animals and humans. Pyroptosis is a proinflammatory form of programmed cell death that is important in response to pathogen infection. The role of pyroptosis in Cp infection and the related mechanisms are still unclear. Here, we reveal that Cp infection induces pyroptosis in macrophages. The inhibition of pyroptosis by disulfiram (DSF) or dimethyl fumarate (DMF) decreases the pathogenicity of Cp in mice and prevents the escape of this pathogen from infected macrophages. In contrast, LPS treatment achieves the opposite results in mice and macrophages infected with Cp. In addition, we revealed that phospholipase D (PLD) is a key virulence factor that induces pyroptosis and subsequently promotes Cp escape and spread in macrophages and confirmed that mutation of the enzyme active site at D66S, G80I, K114N, and G242P weakens PLD-induced pyroptosis. Furthermore, the indispensable role of the NLRP3-GSDMD axis in PLD-induced pyroptosis was confirmed using *Nlrp3* knockout (*Nlrp3*^*−/−*^) and *Gsdmd* knockout (*Gsdmd*^*−/−*^) macrophages. Mechanistically, the PLD of Cp targets mitochondrial sphingomyelins within macrophages, induces cardiolipin externalization to the outer mitochondrial membrane, and releases mtROS, leading to pyroptosis. In conclusion, our data indicate that Cp infection induces mitochondrial dysfunction in macrophages through the secretion of PLD, which targets mitochondrial sphingomyelins and induces NLRP3-GSDMD axis-dependent pyroptosis. Inducing pyroptosis is one of the mechanisms underlying the pathogenesis and transmission of Cp, and inhibiting pyroptosis may be an important strategy for controlling this pathogen.

**Supplementary Information:**

The online version contains supplementary material available at 10.1186/s13567-025-01640-7.

## Introduction

*Corynebacterium pseudotuberculosis* (Cp), belonging to the genus *Corynebacterium* of the phylum *Actinomycetota*, is a zoonotic pathogen that infects livestock, wildlife, and humans [1], of which sheep and goats are the predominant hosts for this pathogen infection [2]. Cp infection results in chronic inflammatory diseases characterized by the formation of abscesses and caseous lymphadenitis (CLA) in the surface lymph nodes or internal organs of infected animals [3, 4] and causes severe economic losses in livestock husbandry because of decreased production performance and the increased number of condemned and downgraded carcasses during meat inspections [5]. Revealing the pathogenic mechanism of Cp infection is essential for preventing these diseases.

As a facultative intracellular bacterium, Cp can invade and survive within macrophages and ultimately kill the infected cells [6]. Phospholipase D (PLD), the key exotoxin of Cp, has been detected in all isolated strains [5]. This enzyme catalyses the hydrolysis of sphingomyelin (SM) [7] and plays a critical role in the persistence and spread of this pathogen within the host [8]. Previous studies have reported that at the initial stage of *M. marinum* infection, the bacterial population expands by first infecting and killing the infected macrophages via apoptosis. Subsequently, the bacterial contents of the apoptotic cells are engulfed by new macrophages [9, 10]. These findings suggest that the infection of macrophages might be a strategy through which intracellular bacteria spreads. However, Cp infection does not lead to the induction of autophagy or apoptosis in macrophages [6]. The mechanism by which Cp induces macrophage death and the role of PLD in this process remain unclear.

Pyroptosis is a proinflammatory form of programmed cell death characterized by the rupture of the cell membrane, the leakage of cellular contents, and the release of inflammatory cytokines such as lactate dehydrogenase (LDH) and IL-1β [11, 12]. Pyroptosis can be activated by a variety of signals, and pyroptosis induced by the canonical inflammasome pathway involves (1) the activation of the NLRP3 inflammasome, resulting in the production of active caspase-1, and (2) the cleavage of gasdermin D (GSDMD) by caspase-1 to generate the N-terminal active fragment (GSDMD-N), which induces pyroptosis [13, 14]. Pyroptosis plays dual roles in the host response to pathogenic infections. Normally, it plays a protective role in pathogen infection, as it promotes the induction of inflammatory responses and prevents intracellular pathogen replication [15, 16]. However, the occurrence of pyroptosis also enables bacteria to escape from infected cells and spread. Some pathogens, such as *Candida albicans* and *Mycobacterium tuberculosis*, can escape from macrophages and further infect other cells by hijacking the pyroptosis pathway [17, 18].

We previously reported that the NLRP3 inflammasome is critical for IL-1β secretion in macrophages infected by Cp [19]. This study further reveals that PLD induces pyroptosis by disrupting mitochondria, activating the NLRP3-GSDMD axis, and inducing plasma membrane rupture facilitated by NINJ1 oligomerization. These findings provide a deeper understanding of the molecular pathways involved in Cp escape and dissemination from host cells.

## Materials and methods

### Mice

C57BL/6 mice (8–10 weeks old) were purchased from Hunan Silaikejingda Experimental Animal Co., Ltd. *Nlrp3*^*−/−*^ mice were kindly provided by Feng Shao from the National Institute of Biological Sciences (NIBS; Beijing, China). Kunming mice (8–10 weeks old) were obtained from the Experimental Animal Center of Southwest Medical University. The mice were housed under controlled conditions (24 ± 2 °C, 55 ± 15% relative humidity) and fed a standard rodent diet. All animal experiments were approved by the Ethics Committee of Southwest University, Chongqing, China (Permit Number: IACUC-20230410-03).

### Bacterial strains, plasmids, and culture conditions

The Cp Xuanhan (XH02), *pld*-deleted (XH02Δ*pld*) [20], complementary PLD (XH02Δ*pld:pld*)-expressing and site mutant PLD (XH02Δ*pld:mpld*)-expressing strains and the plasmids used in this research are detailed in Additional file 1. Cp was cultivated in Luria–Bertani (LB) broth supplemented with 10% foetal bovine serum (FBS) (Biological Industries, 04-001-1ACS) and 0.1% Tween 80 (Solarbio, IT9000) or on LB agar plates supplemented with 5% rabbit blood at 37 °C. To cultivate Cp carrying the pXMJ19 or pXMJ19-derived plasmids, chloramphenicol (10 µg/mL) was added to LB broth or LB agar plates to maintain antibiotic selection pressure.

### Cell culture

J774A.1 macrophages, *Nlrp3* knockout (*Nlrp3*^−/−^) and *Gsdmd* knockout (*Gsdmd*^*−/−*^) J774A.1 cells, and HEK293T cells were maintained in DMEM supplemented with 10% FBS, 17.5 mM D-glucose, 2.5 mM L-alanyl-L-glutamine, 0.5 mM sodium pyruvate, 1 × nonessential amino acids, and 1 × MEM vitamins. Peritoneal macrophages (PMs) from mice were prepared according to a previously described procedure [21] and cultured in RPMI 1640 medium supplemented with 10% FBS.

### Construction of Cp strains with complementary PLD or site-mutant PLD expression

The primers used in this study are listed in Additional file 2. The *pld* gene sequence was amplified from Cp genomic DNA via PCR. Site-directed mutagenesis was performed to generate mutant *pld* (*mpld*) using primers containing mismatches at target sites, as previously described [22]. Both *pld* and *mpld* were cloned and inserted into a modified pXMJ19 vector (with the *lacIq* sequence deleted) to construct the constitutive expression plasmids pXMJ19-*pld* and pXMJ19-*mpld*. These plasmids were then electroporated (parameters: 2.5 kV, 12 ms [23]) into XH02Δ*pld* to generate (i) a complementary PLD-expressing strain (XH02Δ*pld*:*pld*) and (ii) a mutant PLD (D66S, G80I, D112H, K114N, Y151P, or W242P)-expressing strain (XH02Δ*pld*:*mpld*).

### PLD or site-mutated PLD recombinant protein expression and purification

The *pld* or *mpld* sequence was amplified via PCR from pXMJ19-*pld* or pXMJ19-*mpld* and cloned and inserted into the pCold-TF vector, yielding pCold-*pld* or pCold-*mpld*. These constructs were subsequently transformed into *E. coli* BL21 (DE3), after which the bacteria were cultured to the stationary phase, followed by induction with 0.5 mM IPTG at 16 °C for 18 h. The cells were subsequently pelleted by centrifugation and resuspended in ice-cold physiological saline. Recombinant PLD (rPLD) or mutant rPLD (D66S, G80I, D112H, K114N, Y151P, and W242P) expression in the supernatant was verified by SDS‒PAGE, and the proteins were purified on a Ni‒NTA column (TransGene, DP101-01). After purification, the imidazole was removed by dialysis in PBS (4 °C, 12 h), and the proteins were lyophilized for storage.

### Construction of pCMV-*pld* and transfection

The sequence of *pld* was amplified from Cp DNA using the forward primer PLD-F (EcoR Ⅰ) and the reverse primers PLD-FLAG-R, PLD-FLAG2-R and PLD-FLAG3-R (*Xho* Ⅰ). The PCR-amplified product and pCMV vector were digested by *Eco*R Ⅰ (Takara, 1611) and *Xho* Ⅰ (Takara, 1635), followed by ligation to form pCMV-*pld*, which was subsequently transformed into DH5α competent cells. J774A.1 cells were transfected with pCMV-*pld* using PEI (Polysciences, 24765-100) at 37 °C with 5% CO_2_ for varying durations depending on the experimental requirements.

### In vitro infection of macrophages with Cp

J774A.1 cells or PMs were cultured in 6-well culture plates (2 × 10^6^ cells/well for western blot analysis), 12-well culture plates (1 × 10^6^ cells/well for mitochondrial membrane potential and mitochondrial ROS analyses), 48-well culture plates (1 × 10^5^ cells/well for the LDH assay), or 100-mm cell culture dishes (1.5 × 10^7^ cells/dish for transmission electron microscopy observation) and infected with XH02, XH02Δ*pld*, XH02Δ*pld*:*pld* or XH02Δ*pld*:*pld* at a multiplicity of infection (MOI) of 10. One hour after infection, the cells were washed three times with PBS and then treated with cell culture medium supplemented with gentamicin (100 μg/mL) to kill extracellular bacteria, after which the cells were cultured for the specified treatment time.

### RNA sequencing (RNA-seq) and data analysis

PMs were infected with XH02 and XH02Δ*pld* (MOI = 10) for 4 h. Total RNA was extracted using RNAiso Plus (Takara, 9109), and RNA-seq was performed by Shanghai Majorbio Biotechnology Co., Ltd., using an Illumina HiSeq Xten (Illumina, USA). A fold change ≥ 1.5 and a *P* value < 0.05 were calculated and visualized to identify significantly differentially expressed genes using edgeR software. A heatmap of gene groups is presented by the Majorbio I-Sanger Cloud Platform. Gene Ontology (GO) and Kyoto Encyclopedia of Genes and Genomes (KEGG) analyses were performed to determine the functional and biological properties of the genes.

### LDH assay

The release of LDH from Cp-infected or pCMV-*pld*-transfected macrophages was determined using an LDH assay kit (Beyotime, C0017). The formula for determining the amount of LDH released was as follows: LDH release (%) = 100 × (absorbance of treated samples-absorbance of control samples)/(absorbance of maximum enzymatic activity of cells—absorbance of control samples).

### Western blot analysis

Whole-cell lysates were obtained by lysing macrophages with radioimmunoprecipitation assay (RIPA) buffer (Invent, IN-WB001). The total protein concentration was determined using a BCA protein assay kit (Beyotime, P0010). The proteins were separated by SDS‒PAGE and transferred to polyvinylidene fluoride membranes. The membranes were incubated overnight with primary antibodies against GSDMD (Abcam, ab209845), Caspase-1 (AdipoGen, AG-20B-0042), IL-1β (R&D Systems, AF-401-NA), NLRP3 (AdipoGen, AG-20B-0042-C100), or β-actin (Proteintech, 66009-1-Ig). After three washes with PBST, the membranes were incubated with an HRP-labelled secondary antibody (Proteintech, SA00001-1, SA00001-2, SA00001-4) for 1 h. Subsequently, the signals were detected using a BeyoECL Star kit (Beyotime, P0018AS).

### Mitochondrial membrane potential and mitochondrial reactive oxygen species assays

Four hours after Cp infection, the macrophages were washed three times with PBS, the mitochondrial membrane potential was determined using a JC-1 kit (Solarbio, M8650), and mitochondrial reactive oxygen species (mtROS) levels were determined with MitoSOX Red Mitochondrial Superoxide Indicator (Yeasen, 40778ES50). Images of red and green fluorescent macrophages at the same location (for membrane potential) and images of red fluorescent macrophages (for mtROS) were randomly captured with a fluorescence microscope. The fluorescence intensity was analysed using ImageJ software.

### Bacterial escape and spread assays

PMs (2 × 10^6^ cells/well in 6-well plates) or J774A.1 cells (1 × 10^6^ cells/well in 12-well plates) were infected with XH02-*sfGFP* or XH02Δ*pld*-*sfGFP* at an MOI of 1 for 1 h. After three washes with PBS, the macrophages were treated with RPMI1640 (PMs) or DMEM (J774A.1) supplemented with gentamicin (100 μg/mL) for 1 h to kill extracellular bacteria, followed by incubation in antibiotic-free medium (RPMI1640 or DMEM supplemented with 10% FBS) for an additional 10 h.

1. Bacterial invasion: The number of intracellular bacteria at 1 h post-infection was quantified using a plate dilution counting method [19].
2. Bacterial escape: The number of bacteria released into the supernatant at 12 h post-infection was quantified via a plate dilution counting method.
3. Bacterial spread: (i) In PMs, at 12 h post-infection, fluorescence microscopy was used to capture dual-channel images (green fluorescence and bright field). The images were merged (ImageJ), and the proportion of sfGFP-positive PMs was quantified. (ii) In J774A.1 cells, bacterial spread was assessed by flow cytometry at 12 h post-infection, as previously described [24].

### Immunofluorescence

J774A.1 cells or PMs grown on a glass bottom cell culture dish were washed with PBS and fixed with Immunol Staining Fix Solution (Beyotime, P0098). Immunol Staining Blocking Buffer (containing 0.1% Triton Ⅹ-100) was used overnight blocking and permeabilization at 4 °C. The cells were incubated with anti-TOM20 (CST, 42406S) or anti-NINJ1 (Biorbyt, orb3021) antibodies overnight at 4 °C and then incubated with secondary antibodies conjugated with Alexa 488 (Beyotime, P0176) or Alexa 647 (Beyotime, P0191). Following DAPI (Beyotime, P0131) staining, the coverslips were mounted and analysed under a confocal laser scanning microscope (Zeiss LSM800, Germany).

### Quantitative real-time PCR

Total RNA was extracted from Cp or macrophages using RNAiso Plus (Cat#: 9109; Takara) and reverse transcribed using an RT Reagent Kit (Takara, RR037A). qPCR was performed on an Archimed X4 real-time PCR system (Rocgene) using a SYBR premix Ex Taq kit (Takara, RR820A). Relative gene expression was analysed using the 2^−ΔΔCt^ method, with β-actin as the internal control of the macrophages and 16S rRNA as the internal control of Cp.

### Transmission electron microscopy (TEM)

J774A.1 cells or PMs were seeded in a 100-mm cell culture dish (1.5 × 10^7^ cells/dish) and infected with Cp at an MOI of 10 for 1 h. After three washes with PBS, the macrophages were cultured in cell culture medium containing gentamicin (100 μg/mL) for an additional 7 h. The macrophages were fixed with 2.5% (v/v) glutaraldehyde for 12 h at 4 °C and with 1% (w/v) osmium tetroxide at room temperature for 1 h, after which the cells were dehydrated through a graded series of acetone. The dehydrated cells were subsequently infiltrated with acetone-Epon 812 resin mixtures for 4 h and 100% Epon 812 resin overnight. Ultrathin serial sections were collected on copper formvar-coated slot grids, stained with 2% (w/v) uranyl acetate and lead citrate, and visualized under an electron microscope**.**

### CRISPR-Cas9-based generation of knockout cell lines

*Nlrp3*^*−/−*^ and *Gsdmd*^*−/−*^ cell lines derived from J774A.1 cells were generated utilizing CRISPR technology. Single-guide RNAs (sgRNAs) for mouse *Nlrp3* and *Gsdmd* (Additional file 2) were designed with an online ChopChop and then inserted into a lentiCRISPRv2-mCherry vector to produce the plasmids lentiCRISPRv2-mCherry-*Nlrp3* and lentiCRISPRv2-mCherry-*Gsdmd*. After the transfection of plasmids containing guide RNA sequences and the Cas9 gene into HEK293T cells alongside lentivirus helper plasmids (PsPAX2, pMD2. G), and the supernatants were harvested at 48 h post-transfection. The concentrated viral particles were then used to infect J774A.1 cells, followed by flow cytometry-based sorting.

### Mitochondrial fractionation

Mitochondria were extracted with a Mitochondrial Isolation and Protein Extraction Kit (Proteintech, PK10016) as previously reported [25]. Briefly, macrophages were collected in cold PBS and centrifuged at 500 × *g* for 5 min at 4 °C. After the supernatant was removed, the cells were resuspended in 1 mL of buffer A and homogenized on ice by ultrasonication. The homogenates were slowly placed on buffer B and centrifuged at 600 × *g* for 10 min at 4 °C. The supernatants were collected and further centrifuged at 10 000 × *g* for 10 min at 4 °C, after which the resulting mitochondrial pellet was obtained. When needed, mitochondrial protein concentrations were determined using a BCA Protein Assay Kit (Beyotime, P0010).

### Sphingomyelin (SM) assay

Mitochondria extracted from macrophages were subjected to experimental processing and then tested using a Sphingomyelin Assay Kit (Abcam, ab138877), as previously reported [26]. Briefly, mitochondrial samples were added to a black ELISA plate, followed by the addition of SMase working solution and incubation for 1 h. Then, the sphingomyelin determination mixture was added and further incubated for 1 h, after which the fluorescence intensity was measured at Ex/Em = 540/590 nm.

The complete cell membrane was isolated and extracted using a Plasma Membrane Protein Isolation and Cell Fractionation Kit (Invent, SM-005) according to the manufacturer’s instructions [27]. One-fifth of the cell membrane samples were removed and dissolved in RIPA lysis buffer for protein quantification, and the cell membrane concentration was standardized on the basis of the protein quantification results. The sphingomyelin levels in the cell membrane samples were determined using a Sphingomyelin Assay Kit (Abcam, ab138877).

### Mitochondrial cardiolipin (CL) externalization determination

Annexin V has a unique affinity for cardiolipin (CL), and the exposure of CL on mitochondria can be detected by its binding to annexin V [28, 29]. The mitochondria were incubated with MitoTracker Red CMXRos (Beyotime, C1035) for 10 min. After centrifugation, Annexin V-FITC (Beyotime, C1062M) was added, and the samples were incubated for 15 min. The samples were treated with three times the volume of cold PBS and analysed using flow cytometry to detect mitochondrial CL exposure.

### In vivo infection of mice with Cp

To assess the role of pyroptosis in Cp infection, C57BL/6 mice were intraperitoneally injected with DSF (50 mg/kg), DMF (50 mg/kg), or LPS (2 mg/kg), followed by intraperitoneal infection with XH02 (6 × 10^5^ CFU/mouse) for 24 h (for the DSF or DMF groups) or 4 h (for the LPS group), and the mice were monitored every day until 14 days post-infection. Furthermore, wild-type (WT) and *Nlrp3* knockout (*Nlrp3*^*−/−*^) C57BL/6 mice were intraperitoneally injected with XH02 (2 × 10^5^ CFU/mouse). The mice were monitored every day until 14 days post-infection.

To evaluate the effects of PLD and site-mutant PLD on XH02Δ*pld*-induced pyroptosis, Kunming mice were intraperitoneally injected with XH02, XH02, XH02Δ*pld*, XH02Δ*pld:pld*, or XH02Δ*pld:mpld* (8 × 10^6^ CFU/mouse). On the third day post-infection, the serum level of IL-1β in the mice was determined using an ELISA kit (Invitrogen, 88-7013A-88). The mice were monitored every day until 14 days post-infection, and the remaining surviving mice were dissected to observe the pathological changes in their abdominal organs.

### Molecular docking

The 2D structure of SM was drawn in ChemDraw and imported into ChemDraw 3D for energy minimization using the MM2 module to obtain the lowest energy conformation. The 3D structure of the PLD was obtained from the I-TASSER database. Water molecules and original ligands were removed using PyMOL, and the protein was prepared in Mgtools 1.5.6 by adding hydrogens, calculating charges, and merging nonpolar hydrogens. The resulting structure was saved in PDB format. Ligand–receptor docking was performed with Autodock Vina 1.1.2 and visualized using PyMOL. Protein‒ligand interactions were analysed with LigPlot + , which generated 2D schematic representations of intermolecular interactions, including hydrogen bonds (dashed lines) and hydrophobic contacts, on the basis of the input PDB file.

### Statistical analysis

The quantitative variables are expressed as the mean ± standard error. All analyses were performed using GraphPad Prism software. Statistical significance between two groups was analysed by Student’s *t* test. A *P* value < 0.05 was considered significant.

## Results

### Cp infection induces pyroptosis in macrophages

**Figure 1:**
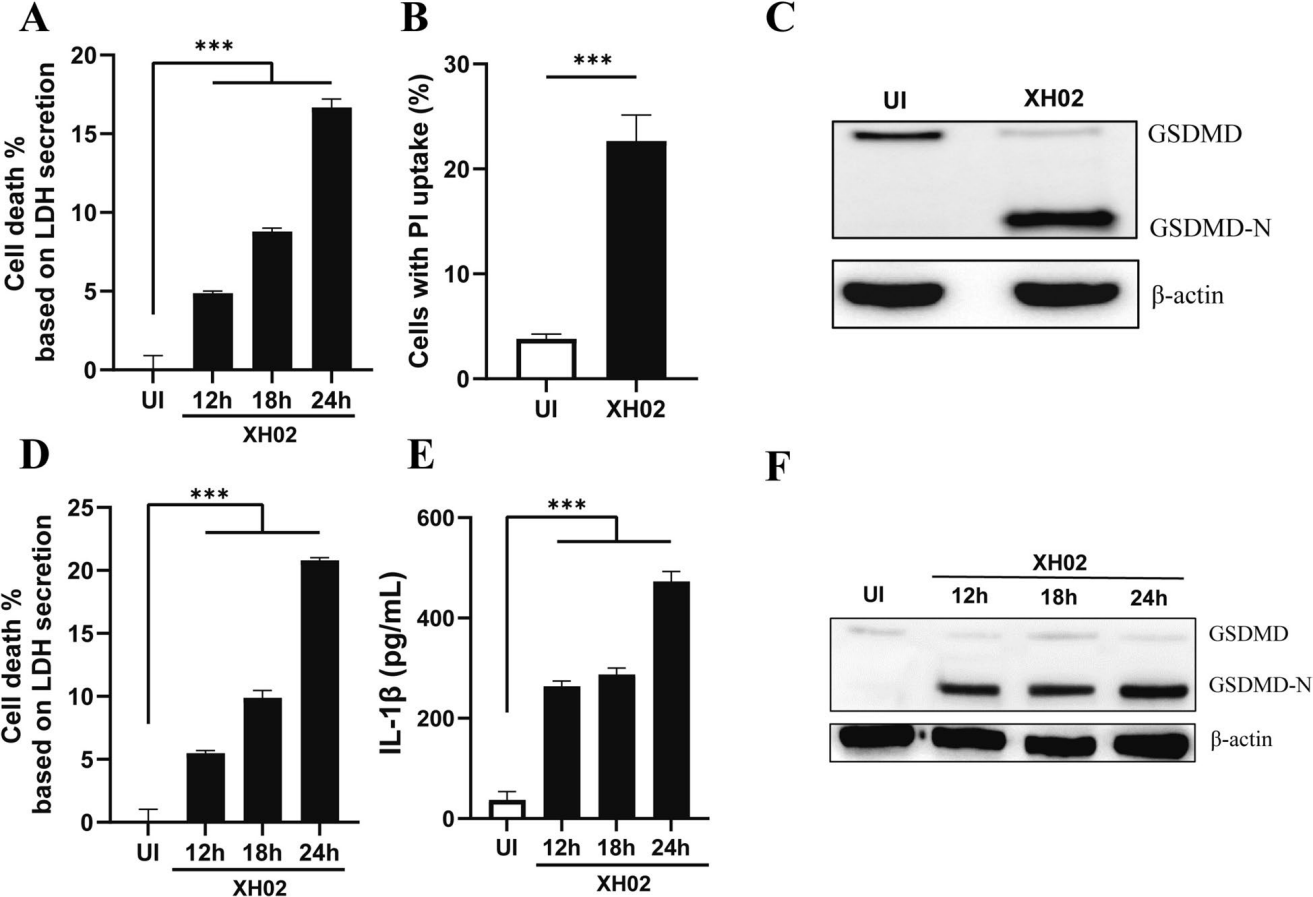
**Cp infection induces macrophage pyroptosis. A** and** D** LDH release by murine peritoneal macrophages (PMs) (A) and J774A.1 macrophages (D) infected with XH02 (MOI = 10) for 12, 18, and 24 h was detected (*n* = 3; n refers to independent biological replicates; the same applies below). **B** The percentage of PI-positive PMs infected with XH02 (MOI = 10) for 12 h was quantified by counting randomly chosen visual fields (*n* = 9). **E** IL-1β secretion by J774A.1 cells infected with XH02 (MOI = 10) for 12, 18, and 24 h was determined (*n* = 3). **C** and **F** Expression of GSDMD/GSDMD-N in XH02-infected PMs (MOI = 10) for 24 h (C) or in XH02-infected J774A.1 (MOI = 10) for 12, 18, and 24 h. A, B, D, and E are representative of three independent experiments. UI, uninfected; error bars represent the SEM; statistical significance was determined by a two-tailed Student’s *t* test: ***P < 0.001.

To determine whether pyroptosis occurred in Cp-infected macrophages, we examined (1) LDH release and PI uptake in the cells, both of which are indicators of cell membrane integrity [30], and (2) the cleavage of GSDMD to GSDMD-N, which is an executor of pyroptosis [13, 14]. We found that LDH release, PI uptake, and GSDMD cleavage were increased in XH02-infected PMs compared with uninfected cells (Figures 1A–C), and similar results were observed in XH02-infected J774A.1 macrophages (Figures 1D–F). These results collectively indicate that Cp infection induces pyroptosis in macrophages.

### Pyroptosis contributes to Cp infection in mice and facilitates Cp escape from infected macrophages

**Figure 2:**
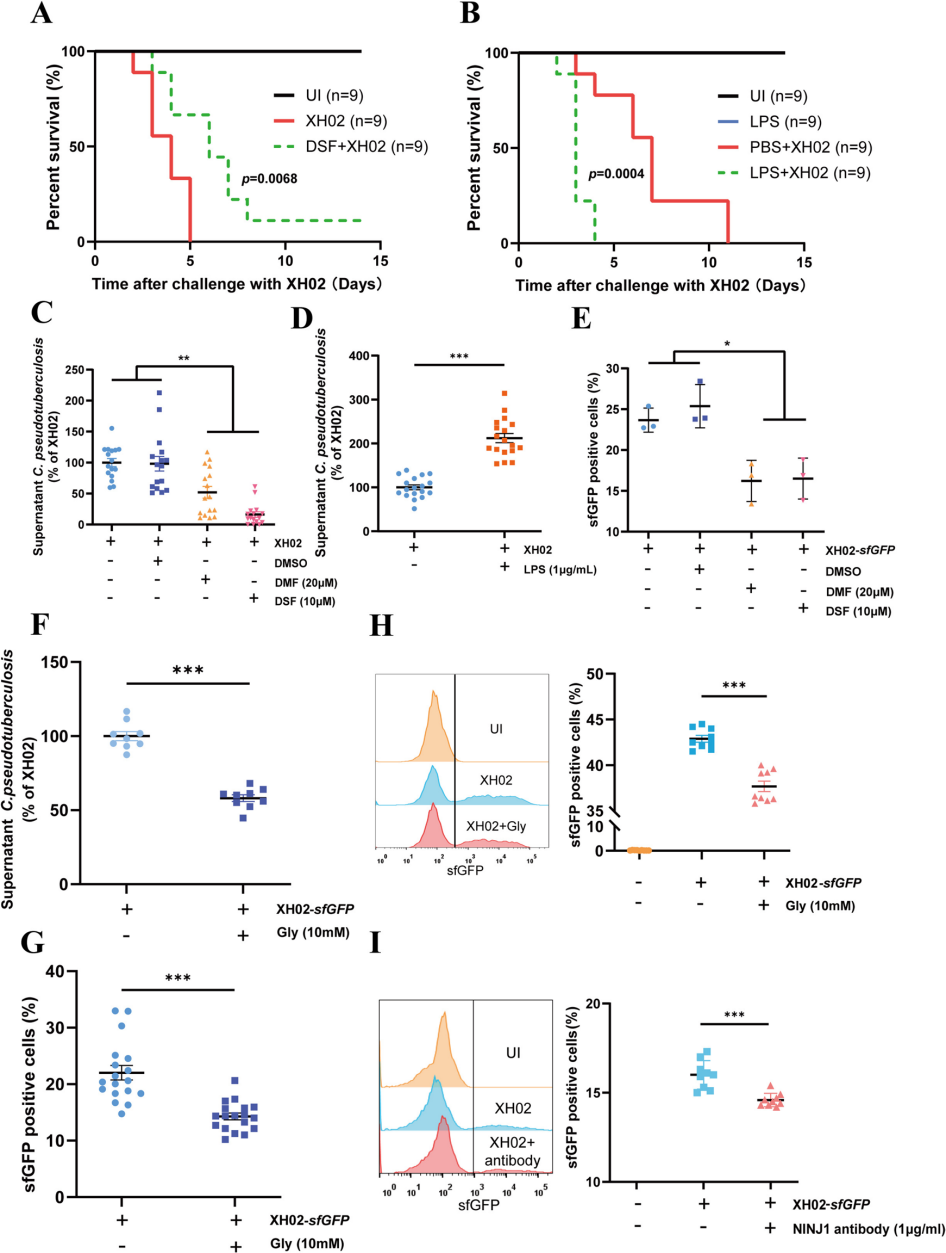
**Pyroptosis contributes to Cp infection in mice and facilitates Cp escape from macrophages. A** and **B** C57BL/6 mice were injected with DSF (50 mg/kg) at 24 and 4 h (**A**) or with LPS (2 mg/kg) at 4 h (**B**) before an intraperitoneal challenge with XH02 (6 × 10^5^ CFU/mouse) and monitored for 14 d (*n* = 9). **C-E** PMs were pretreated with DMF (20 μM), DSF (10 μΜ) or LPS (1 μg/mL) for 1 h, followed by bacterial escape (**C**, *n* = 16; **D**, *n* = 18) and spread assays (**E**) (*n* = 3). **F‒G** PMs were treated with Gly (10 mM), followed by bacterial escape (**F**) (*n* = 9) and spread (**G**) (*n* = 18) assays. **H-I** J774A.1 cells were treated with Gly (10 mM) (**H**) (*n* = 9) or NINJ1 antibody (1 μg/mL) (**I**) (*n* = 9), after which the bacterial spread was measured by flow cytometry. C, D, and F to I are pooled from three independent experiments. E is representative of three independent experiments. The error bars represent the SEMs. Statistical significance was determined by a two-tailed Student’s *t* test: ***P < 0.001, **P < 0.01, *P < 0.05.

To investigate the role of pyroptosis in Cp infection, we treated mice with disulfiram (DSF), an inhibitor of pyroptosis that inhibits the pore-forming activity of GSDMD [31], and dimethyl fumarate (DMF), another type of pyroptosis inhibitor that prevents NLRP3 inflammasome activation [32], and challenged them with XH02. We found that treatment with DSF and DMF increased the survival of XH02-infected mice (Figure 2A and Additional file 3). Furthermore, the survival of *Nlrp3*^*−/−*^ mice was significantly greater than that of wild-type (WT) mice after XH02 infection (Additional file 3). However, treatment of mice with LPS, a molecule that primes NLRP3 inflammasome activation and increases caspase-1 activation [33], significantly accelerated the death of mice infected with XH02 (Figure 2B). These results indicate that pyroptosis contributes to Cp infection in mice.

Pyroptosis typically plays a protective role in the host by preventing pathogen replication [17]; however, it can also enable some pathogens to escape from macrophages and spread [17, 18]. We found that DSF or DMF treatment decreased PI uptake, IL-1β secretion, and GSDMD cleavage in PMs after XH02 infection (Additional file 4). The degree of intracellular pathogen escape from XH02-infected PMs treated with DSF or DMF to the culture medium decreased (Figure 2C), whereas the escape of XH02 from the infected PMs increased when LPS was used to induce pyroptosis (Figure 2D). The spread of bacteria has been extensively studied through the application of flow cytometry [24]. We next evaluated the proportion of infected macrophages using sfGFP-labelled XH02 and found that the spread of XH02 was reduced in macrophages treated with DSF or DMF (Figure 2E). The transmembrane protein ninjurin-1 (NINJ1) is the common executioner of plasma membrane rupture (PMR) in pyroptosis [16], and glycine (Gly) effectively inhibits PMR in cells undergoing pyroptosis by blocking the oligomerization of NINJ1 [34]. We observed that the release of LDH, the escape of XH02 from the infected macrophages to the culture medium, and the spread of XH02 were reduced in the infected PMs treated with Gly (Figures 2F and G and Additional file 4). Similarly, the spread of XH02 in J774A.1 cells decreased after treatment with anti-Gly or anti-NINJ antibodies (Figures 2H and I). These results indicated that pyroptosis facilitates the escape of Cp from infected macrophages and promotes the spread of this pathogen.

### PLD promotes Cp escape from infected macrophages through the induction of pyroptosis

**Figure 3:**
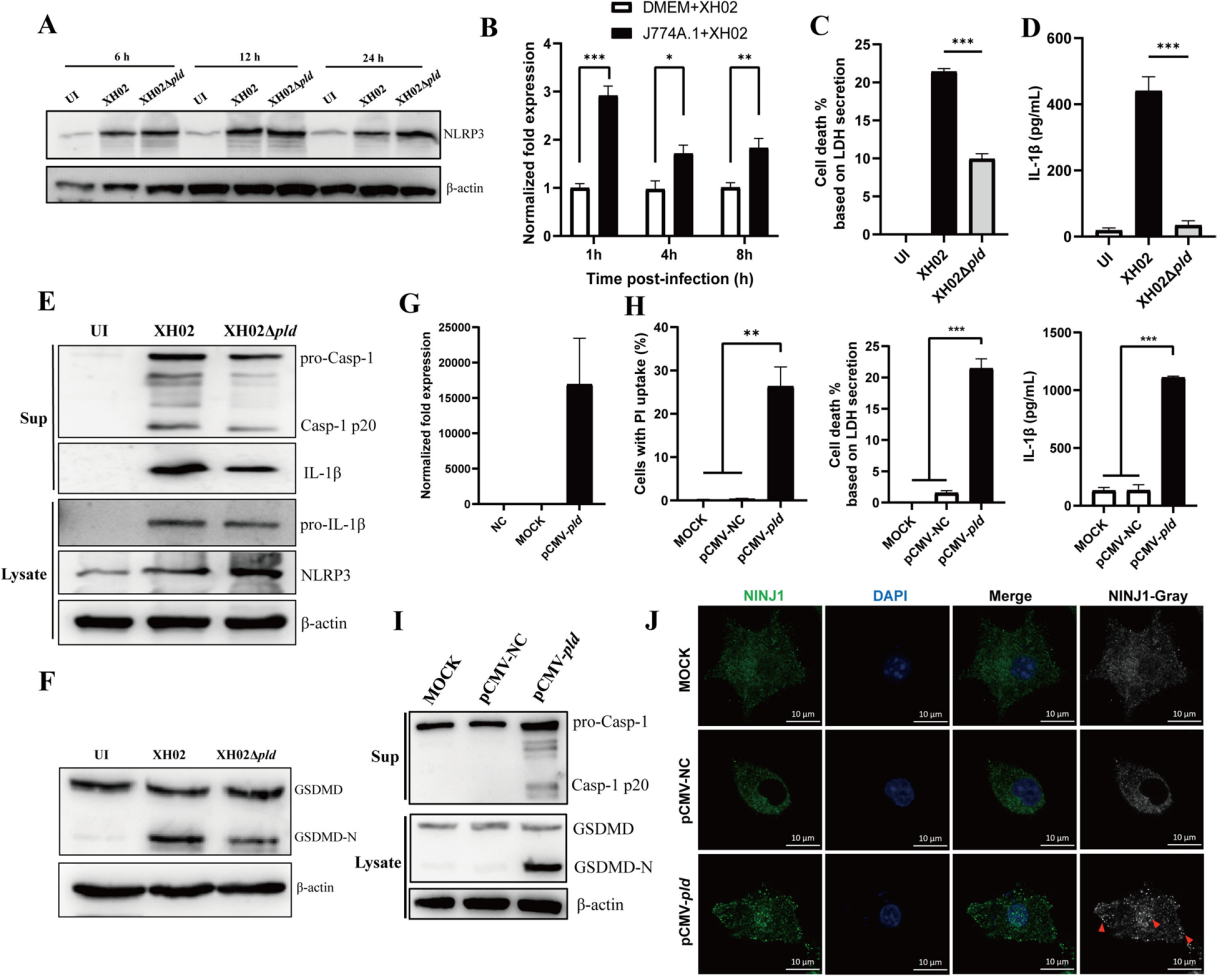
**PLD induces macrophage pyroptosis. A** NLRP3 expression in PMs treated with the supernatant of XH02 cells cultured in RPMI 1640 supplemented with 10% FBS for 6, 12, and 24 h. (B) The expression of *pld* in XH02 cells within J774A.1 cells or cultured in DMEM was measured by qPCR (*n* = 6). **C–D** J774A.1 cells were infected with XH02 or XH02Δ*pld* (MOI = 10) for 12 h. LDH release (C) and IL-1β levels (D) in the supernatants were detected (*n* = 3). **E** Expression of NLRP3, Caspase-1, and IL-1β in PMs infected with XH02 (MOI = 10) for 24 h. **F** Expression of GSDMD in J774A.1 cells infected with XH02 or XH02Δ*pld* (MOI = 10) for 24 h. **G–H** J774A.1 cells were transfected with pCMV*-pld* for 12 h, and the expression of *pld* was measured by qPCR (*n* = 6), and the proportion of PI-uptake cells, LDH release, and IL-1β secretion were measured (*n* = 3). **I** Expression of GSDMD and caspase-1 in J774A.1 cells transfected with pCMV-*pld* for 12 h; untreated cells served as mock controls. **J** Immunofluorescence of J774A.1 cells transfected with pCMV-*pld* for 12 h. The cells were stained with anti-NINJ1 antibodies (green) and DAPI (blue). Images were obtained via laser confocal microscopy. The greyscale images show merged channels, with red arrows indicating NINJ1 oligomerization. C, D, and H are representative of three independent experiments. The error bars represent the SEMs. Statistical significance was determined by two-tailed Student’s *t* test: ***P < 0.001, **P < 0.01, *P < 0.05, ns ≥ 0.05.

Phospholipase D (PLD) is crucial for the pathogenicity and spread of Cp [8, 20]*.* We conducted RNA-seq on macrophages infected with XH02 and XH02Δ*pld*, with three independent biological replicates in each group. Compared with those in the uninfected group, a large number of genes in macrophages were significantly upregulated after XH02 infection, suggesting a marked response of macrophages to Cp infection (Additional file 5). We identified 738 differentially expressed genes in XH02-infected macrophages compared with the XH02Δ*pld* infected cells, including 586 upregulated genes and 152 downregulated genes, by setting an absolute fold change ≥ 1.5 and a *P* value < 0.05 as thresholds. Pyroptosis-related genes, including *Irf1*, *Cmpk2*, *Nlrp3*, *Gsdmd*, and *Il-1β*, were upregulated in XH02-infected macrophages compared with uninfected cells (Additional file 5). Compared with those in XH02-infected macrophages, the mRNA levels of *Irf1*, *Cmpk2*, *Gsdmd*, and *Il-1β*, but not *Nlrp3*, were decreased in XH02Δ*pld*-infected cells, as confirmed by qPCR analysis (Additional file 5). This finding is also consistent with the RNA-seq results, indicating that the degree of pyroptosis-related gene upregulation in macrophages infected with XH02Δ*pld* is far lower than that in those infected with XH02. In addition, incubation of PMs with the supernatant of Cp cultured in RPMI 1640 supplemented with 10% FBS resulted in upregulated NLRP3 expression with independent of *pld* deletion (Figure 3A). Furthermore, the expression of *pld* was significantly greater in XH02 macrophages than in bacteria cultured in DMEM (Figure 3B), suggesting that PLD plays a specific role in the process of Cp infection within macrophages. We further assessed the difference in pyroptosis between macrophages infected with XH02 and XH02Δ*pld* and reported that LDH release and IL-1β secretion were lower in XH02Δ*pld*-infected macrophages than in XH02-infected cells (Figures 3C and D). In addition, the cleavage of pro-Caspase 1 and GSDMD in XH02Δ*pld*-infected macrophages was lower than that in XH02-infected cells (Figures 3E and F). Furthermore, we observed more pronounced oligomerization of NINJ1 induced by XH02 than by XH02Δ*pld* in macrophages (Additional file 6).

We next expressed PLD in macrophages by transfection of pCMV-*pld* (Figure 3G) and found that the intracellular expression of PLD increased the PI uptake, LDH release, and IL-1β secretion of the macrophages (Figure 3H). Additionally, the levels of Caspase-1 p20 and GSDMD-N were increased in pCMV-*pld*-transfected macrophages (Figure 3I). Furthermore, the expression of PLD within macrophages also induces oligomerization of NINJ1 (Figure 3J). These findings indicate that PLD induces pyroptosis accompanied by PMR.

**Figure 4:**
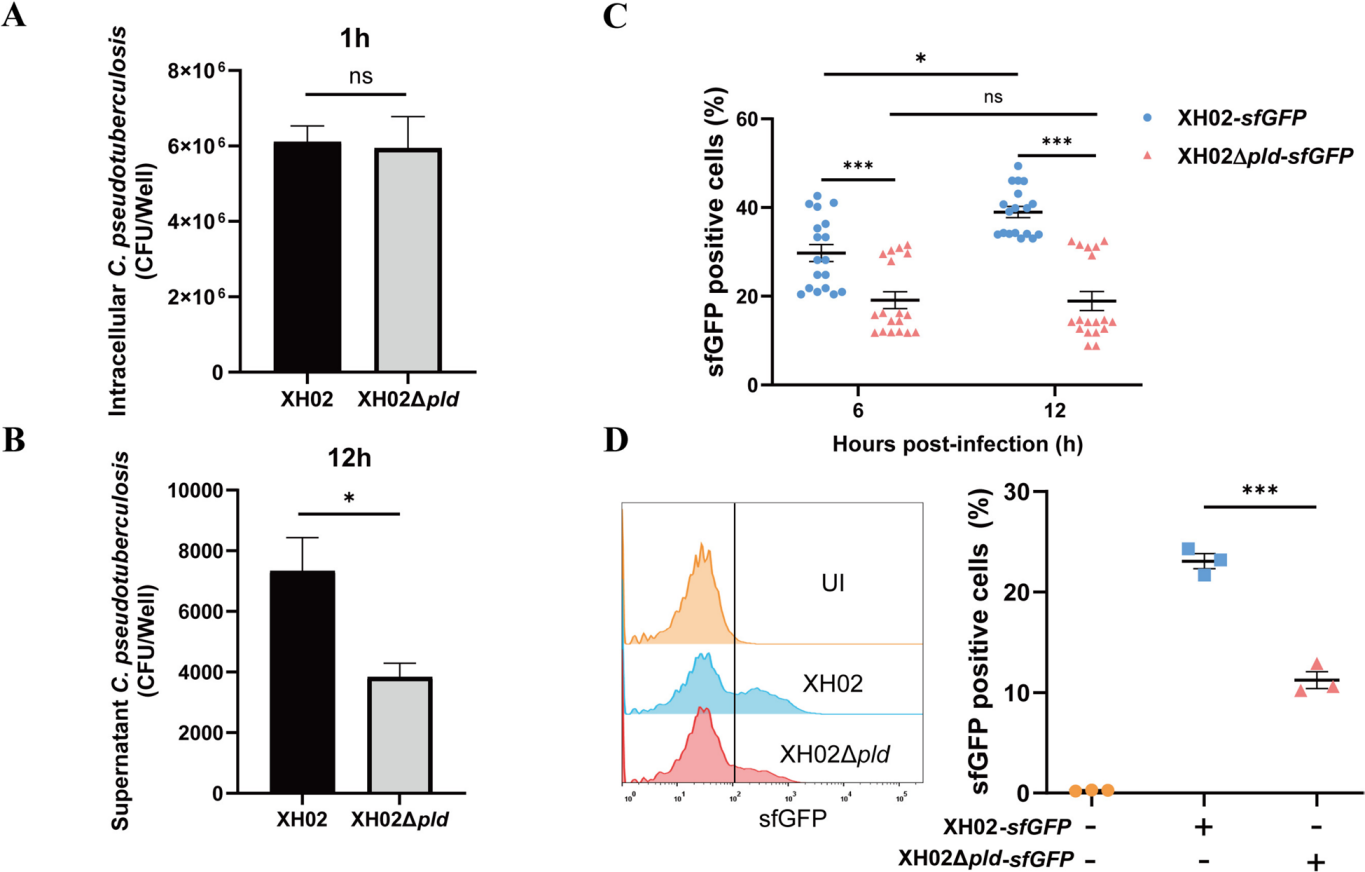
**PLD promotes Cp escape from infected macrophages. K–L** PMs were infected with XH02 or XH02Δ*pld* (MOI = 1), and the number of Cp invading PMs at 1 h (**A**) and escaping into the supernatant from PMs at 12 h (**B**) was determined (*n* = 3). **C** PMs were infected with XH02-*sfGFP* or XH02Δ*pld*-*sfGFP* (MOI = 1) for 6 and 12 h, after which a bacterial spread assay was performed (*n* = 18). **D** J774A.1 cells were infected with XH02-*sfGFP* or XH02Δ*pld*-*sfGFP* (MOI = 1) for 12 h, followed by bacterial spread analysis using flow cytometry (*n* = 3). A, B, and D are representative of three independent experiments. C is pooled from three independent experiments. The error bars represent the SEMs. Statistical significance was determined by two-tailed Student’s *t* test: ***P < 0.001, **P < 0.01, *P < 0.05, ns ≥ 0.05. Statistical significance was determined by two-tailed Student’s *t* test: ***P < 0.001, *P < 0.05, ns ≥ 0.05.

PLD is involved in the spread of Cp within the host [8]. To determine the impact of PLD on the escape and spread of Cp in infected macrophages, we analysed the infection of macrophages by XH02-sfGFP and XH02Δ*pld*-sfGFP. No significant difference in the intracellular bacterial load was detected between XH02-infected and XH02Δ*pld*-infected macrophages at 1 h post-infection (Figure 4A), indicating that PLD is not involved in the replication of Cp in macrophages. However, the number of Cp that escaped into the supernatant from XH02-infected macrophages was significantly greater than that from XH02Δ*pld*-infected cells at 12 h post-infection (Figure 4B). Furthermore, the proportion of XH02-*sfGFP-*infected macrophages was considerably greater than that of XH02Δ*pld*-*sfGFP*-infected PMs (Figure 4C), and a similar result was observed between XH02-*sfGFP-* and XH02Δ*pld*-*sfGFP*-infected J774A.1 cells by flow cytometry analysis (Figure 4D). These data indicate that PLD is involved in the escape of Cp from infected macrophages and the spread of Cp to other macrophages. In summary, the above results demonstrate that PLD promotes Cp escape and spread through the induction of pyroptosis in macrophages.

### PLD is critical for Cp infection-induced mitochondrial dysfunction in macrophages

**Figure 5:**
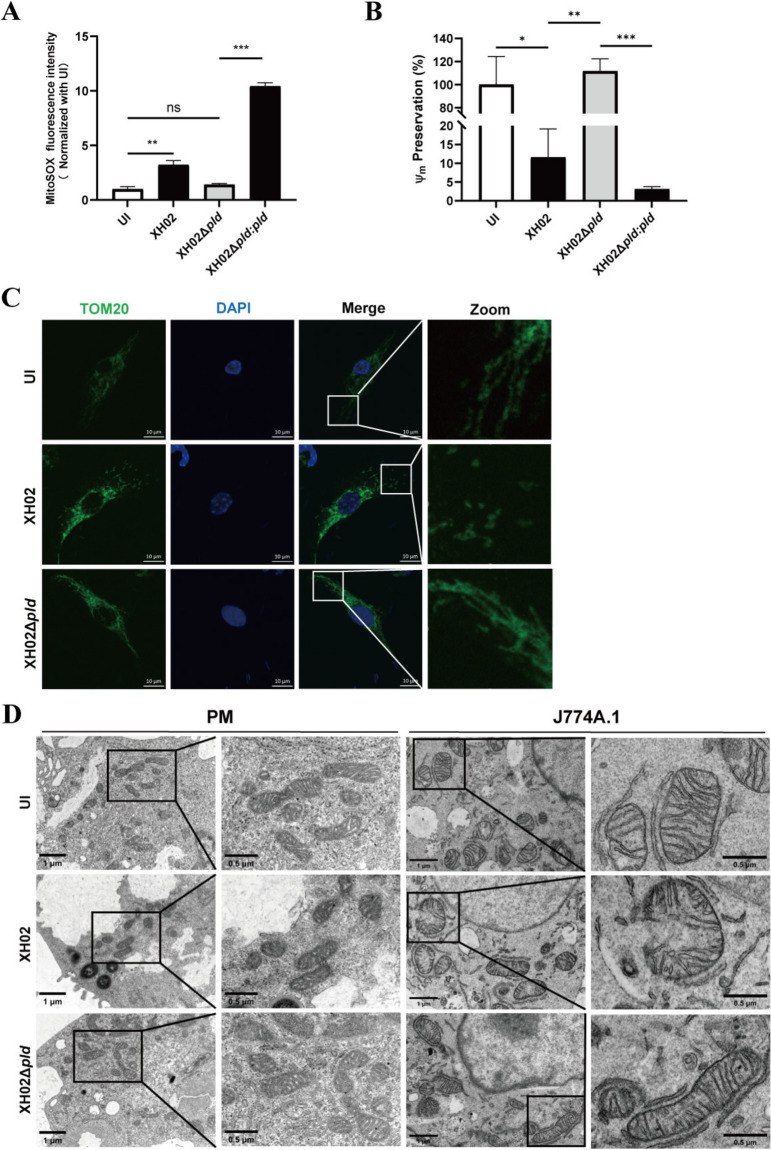
**PLD is critical for mitochondrial dysfunction caused by Cp infection in macrophages. A‒B** PMs were infected with XH02, XH02Δ*pld*, or XH02Δ*pld*:*pld* (MOI = 10) for 4 h. After mitoSOX staining (**A**) or JC-1 staining (**B**), the fluorescence intensity was analysed (*n* = 3). **C** J774A.1 cells were infected with XH02 or XH02Δ*pld* (MOI = 10) for 12 h. Images of anti-TOM20 (green)- and DAPI (blue)-stained macrophages were taken by laser confocal microscopy. **D** Ultrastructures of PMs and J774A.1 cells infected with XH02 or XH02Δ*pld* (MOI = 10) for 8 h. A and B are representative of three independent experiments. UI indicates uninfected cells. The bar indicates 0.5 or 1 μm. The error bars represent the SEMs. Statistical significance was determined by two-tailed Student’s *t* test: ***P < 0.001, **P < 0.01, *P < 0.05, ns ≥ 0.05.

The mitochondrial reactive oxygen species (mtROS) level, mitochondrial membrane potential, and mitochondrial network structure can be used to evaluate mitochondrial dysfunction [35]. We found that compared with uninfected macrophages, XH02- but not XH02Δ*pld*-infected macrophages had significantly increased mtROS levels and decreased mitochondrial membrane potential, and compared with uninfected macrophages, and XH02Δ*pld:pld* infection resulted in a significant increase in mtROS levels and a decrease in mitochondrial membrane potential in macrophages compared with XH02Δ*pld*-infected cells (Figures 5A and B). Furthermore, XH02 but not XH02Δ*pld* led to a more disorganized mitochondrial network structure (Figure 5C). XH02 but not XH02Δ*pld* infection resulted in a condensed mitochondrial matrix in PMs (Figure 5D), suggesting a reduction in mitochondrial membrane potential [36]. We also found that XH02 but not XH02Δ*pld* disrupted cristae and even ruptured mitochondria in J774A.1 cells (Figure 5D). These results indicate that PLD is critical for mitochondrial dysfunction caused by Cp infection in macrophages.

### Mitochondrial dysfunction is involved in Cp-induced pyroptosis

**Figure 6:**
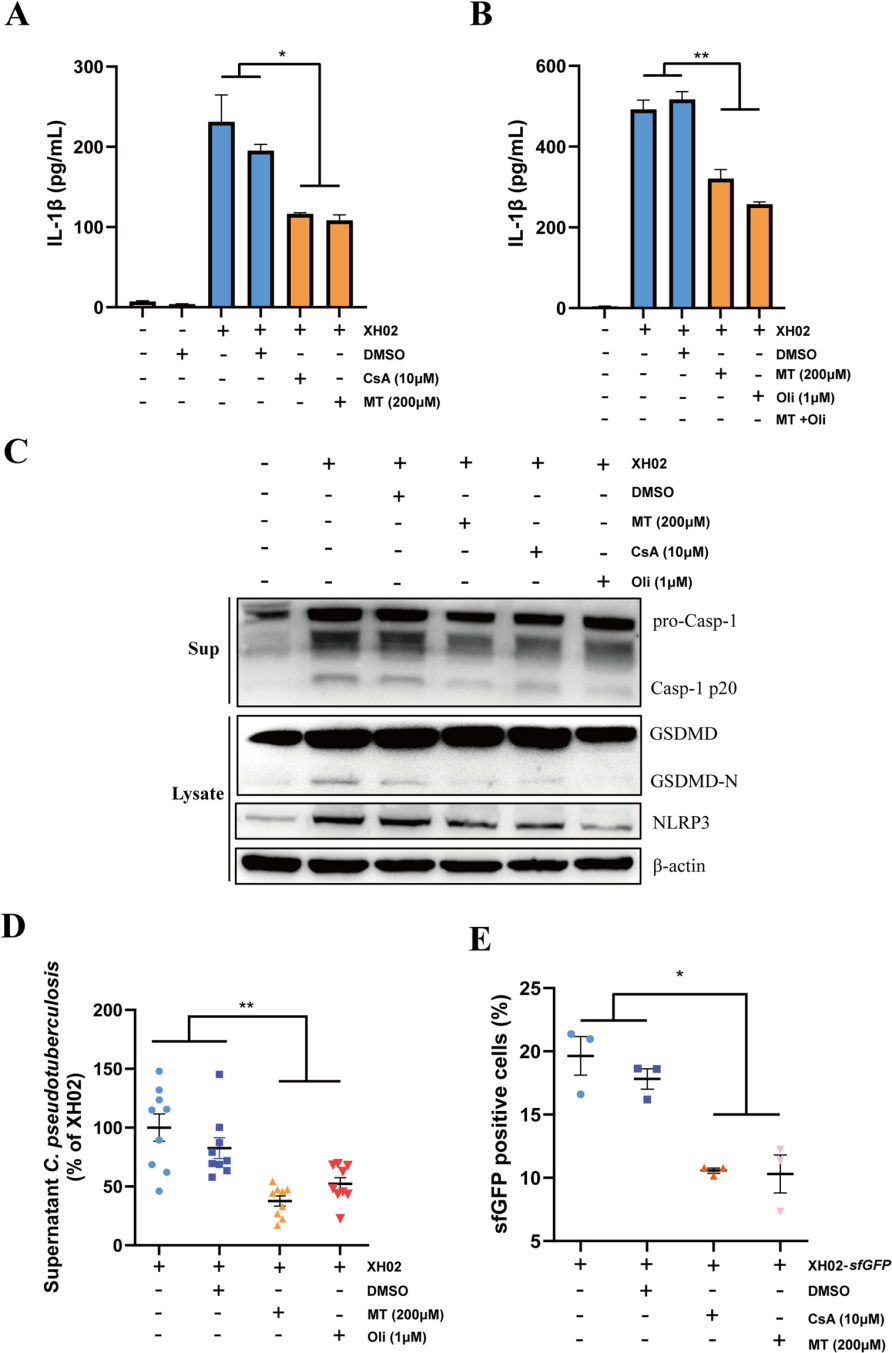
**Mitochondrial dysfunction is involved in Cp-induced pyroptosis. A‒B** PMs were treated with CsA (10 μM), MT (200 μM), or Oli (1 μM) for 1 h and infected with XH02 (MOI = 10) for 12 h. IL-1β secretion in the supernatants was measured (*n* = 3). **C** Expression of NLRP3, GSDMD and Caspase-1 in PMs pretreated with MT (200 μM), Oli (1 μM) or CsA (10 μM) for 1 h and then infected with XH02 (MOI = 10) for 24 h. **D** PMs were pretreated with CsA (10 μM) or MT (200 μM) for 1 h. The number of Cp cells that escaped into the supernatant from the PMs at 12 h was determined (*n* = 9). **E** PMs were pretreated with CsA (10 μM) or MT (200 μM) for 1 h. Then, bacterial spread assays were performed at 12 h post XH02-*sfGFP* infection (MOI = 10) (*n* = 3). A, B, and E are representative of three independent experiments. D is pooled from three independent experiments. The error bars represent the SEMs. Statistical significance was determined by two-tailed Student’s *t* test: **P < 0.01, *P < 0.05.

To determine the role of mitochondrial damage in pyroptosis induced by Cp infection, we used Cyclosporin A (CsA), an inhibitor of mitochondrial permeability transition pore (mPTP) opening [37]; Mito-TEMPO (MT), a mitochondrion-targeted antioxidant that eliminates mtROS [38]; and Oligomycin (Oli), which inhibits mitochondrial complex V (ATP synthase) and blocks the activation of the NLRP3 inflammasome [38, 39]. IL-1β secretion was reduced in XH02-infected macrophages treated with CsA, MT, and Oli (Figures 6A and B). In addition, treatment of macrophages with CsA, MT, and Oli led to decreases in the levels of NLRP3, Caspase-1 p20, and GSDMD-N after Cp infection (Figure 6C). These results suggest that inhibition of mPTP opening and mtROS or ATP generation attenuates NLRP3 activation and pyroptosis in macrophages infected with Cp. Furthermore, treatment of macrophages with CsA or MT decreased the amount of Cp that escaped from the infected macrophages (Figure 6D) and decreased the Cp load in the infected macrophages (Figure 6E).

### PLD induces macrophage pyroptosis through the NLRP3-GSDMD axis

**Figure 7:**
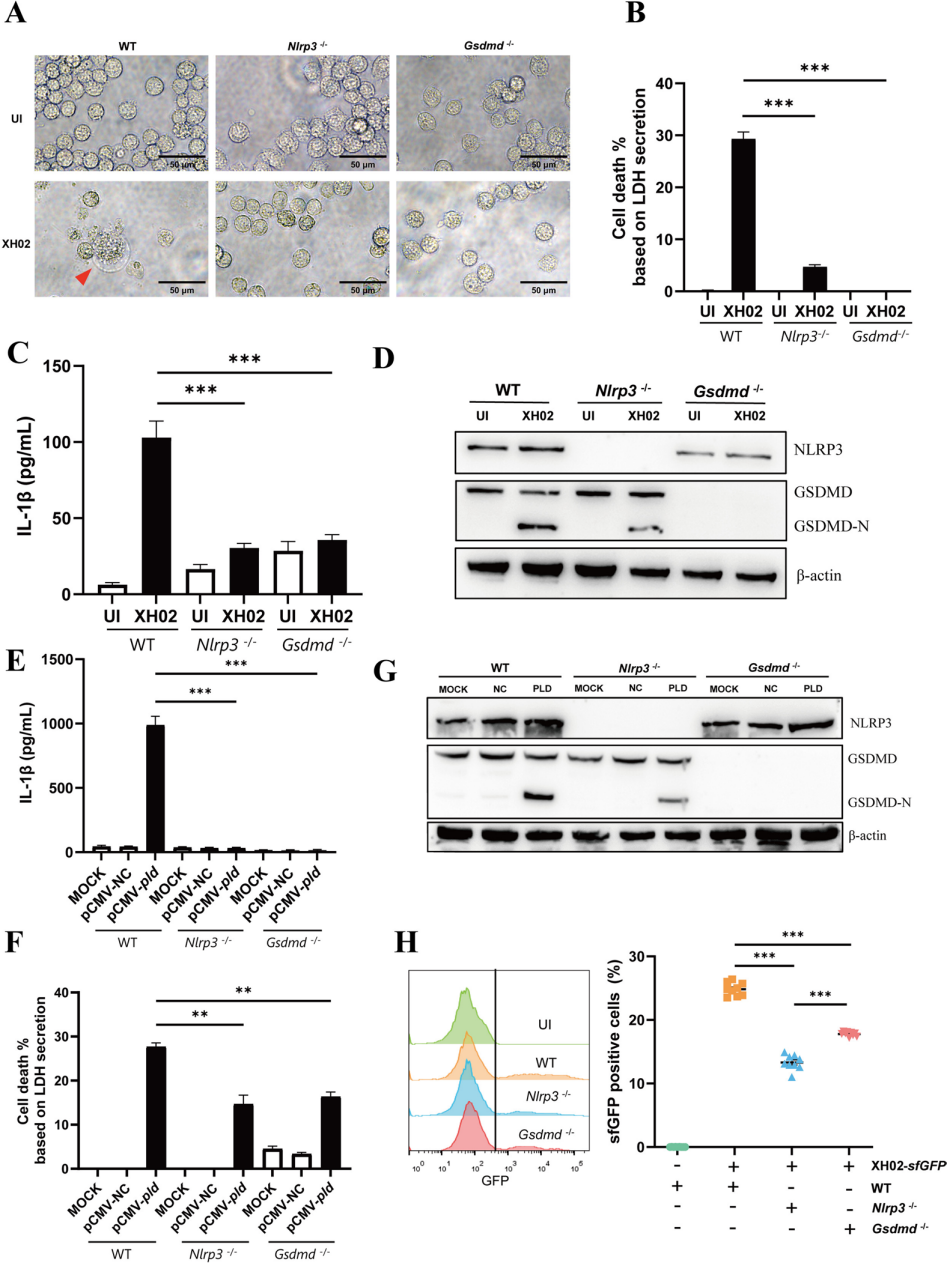
**PLD induces macrophage pyroptosis through the NLRP3-GSDMD axis. A** Representative images of WT, *Nlrp*3^−/−^ and *Gsdmd*^−/−^ J774A.1 cells infected with XH02 (MOI = 10) for 12 h. The red triangle indicates a pyroptotic cell. **B‒C** WT, *Nlrp*3^−/−^ and *Gsdmd*^−/−^ J774A.1 cells were infected with XH02 (MOI = 10) for 12 h, after which LDH release (**B**) and IL-1β secretion (**C**) in the supernatant were measured (*n* = 3). **D** Expression of NLRP3 and GSDMD in WT, *Nlrp*3^−/−^ and *Gsdmd*^−/−^ J774A.1 cells infected with XH02 (MOI = 10) for 24 h. **E–F** WT, *Nlrp*3^−/−^ and *Gsdmd*^−/−^ J774A.1 cells were transfected with pCMV-*pld* for 12 h, and IL-1β secretion (**E**) and LDH release (**F**) in the supernatants were detected (*n* = 3). **G** Expression of GSDMD, NLRP3, and Caspase-1 in WT, *Nlrp*3^−/−^ and *Gsdmd*^−/−^ J774A.1 cells transfected with pCMV-*pld* for 12 h. **H** WT, *Nlrp*3^−/−^ and *Gsdmd*^−/−^ J774A.1 cells were infected with XH02-*sfGFP* (MOI = 1) for 12 h. The bacterial spread assay was performed by flow cytometry (*n* = 9). B, C, E, and F are representative of three independent experiments. H was pooled from three independent experiments. The error bars represent the SEMs. Statistical significance was determined by two-tailed Student’s *t* test: ***P < 0.001, **P < 0.01.

We previously reported that the NLRP3 inflammasome is critical for IL-1β secretion by Cp-infected macrophages [19]. To further confirm whether NLRP3 or GSDMD are indispensable for pyroptosis induced by Cp infection and intracellular PLD expression, *Nlrp3* knockout (*Nlrp3*^−/−^) and *Gsdmd* knockout (*Gsdmd*^*−/−*^) strains derived from wild-type J774A.1 macrophages (WT) were constructed using CRISPR technology. Unlike the XH02-infected WT cells, which exhibited numerous small vesicles, cell swelling, and leakage of contents, the infected *Nlrp3*^−/−^ and *Gsdmd*^*−/−*^ cells maintained their original morphology (Figure 7A). Upon XH02 infection, LDH release and IL-1β secretion were significantly lower in *Nlrp3*^−/−^ and *Gsdmd*^*−/−*^ cells than in WT cells (Figures 7B and C). The level of GSDMD-N in XH02-infected *Nlrp3*^−/−^ J774A.1 cells was lower than that in XH02-infected WT cells (Figure 7D), potentially because of the activation of pyroptosis through the nonclassical pathway by Cp within the cells, leading to the induction of other inflammatory caspases that resulted in the cleavage of GSDMD [40]. In addition, LDH release and IL-1β secretion were lower in pCMV-*pld*-transfected *Nlrp3*^−/−^ and *Gsdmd*^*−/−*^ cells than in pCMV-NC-transfected WT cells (Figures 7E and F). After pCMV-*pld* transfection, the level of GSDMD-N in *Nlrp3*^−/−^ J774A.1 cells was lower than that in WT cells (Figure 7G). Furthermore, compared with that in WT cells, the spread of Cp in *Nlrp3*^−/−^ or *Gsdmd*^*−/−*^ J774A.1 cells was attenuated (Figure 7H), similar to the results of Cp spreading in DMF- or DSF-treated macrophages shown in Figure 2E. These data confirmed that the NLRP3-GSDMD axis is involved in PLD-induced pyroptosis.

### Multiple enzyme active sites are crucial for PLD to induce pyroptosis

**Figure 8:**
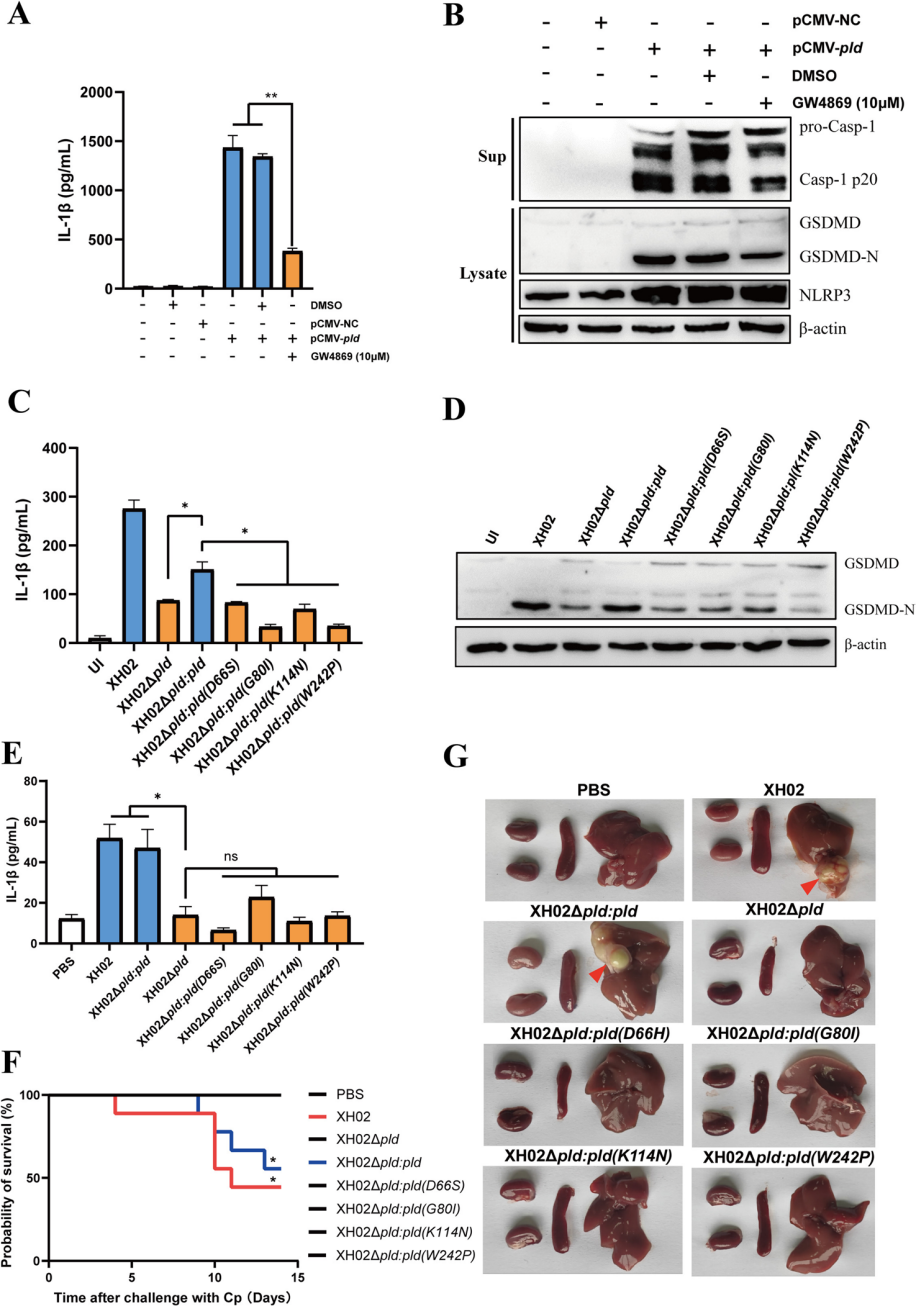
**Multiple enzyme active sites are crucial for PLD to induce pyroptosis. A** J774A.1 cells were treated with GW4869 (10 μM) and transfected with pCMV-*pld*. IL-1β secretion in the supernatant was measured (*n* = 3). **B** Expression of GSDMD, NLRP3, and Caspase-1 in J774A.1 cells transfected with pCMV-*pld* and then treated with GW4869 (10 μM) for 12 h. **C** PMs were infected with XH02, XH02Δ*pld*, XH02Δ*pld*:*pld*, or XH02Δ*pld*:*mpld* (MOI = 10) for 12 h. IL-1β secretion in the supernatant was measured (*n* = 3). D Expression of GSDMD in PMs infected with XH02, XH02Δ*pld*, XH02Δ*pld*:*pld*, and XH02Δ*pld*:*mpld* (MOI = 10) for 24 h. **E–G** Kunming mice were intraperitoneally challenged with XH02, XH02Δ*pld*, and XH02Δ*pld*:*pld*, XH02Δ*pld*:*mpld* (8 × 10^6^ CFU/mouse), the serum IL-1β levels of the mice on the 3rd day post-infection were measured (*n* = 3) (**E**), the survival curve of the *Cp-*infected mice was monitored for 14 days (*n* = 9) (**F**), and the gross lesions in the kidneys, spleen and liver were observed at 14 days post-infection (**G**). A and C are representative of three independent experiments. The error bars represent the SEMs. Statistical significance was determined by two-tailed Student’s *t* test: **P < 0.01, *P < 0.05, ns ≥ 0.05.

The PLD of Cp promotes the hydrolysis of ester bonds in sphingomyelins (SMs) in mammalian cell membranes [41]. We found that treatment with GW4869, a potent inhibitor of SMs that targets the inhibition of neuromycin formation during the process of sphingomyelin hydrolysis [42], significantly decreased IL-1β secretion in pCMV-*pld*-transfected macrophages (Figure 8A) and reduced pro-Caspase-1 and GSDMD cleavage (Figure 8B), suggesting that enzyme activity is associated with PLD-induced pyroptosis.

Through I-TASSER analysis, H44, N66, G80, N112, K114, and W242 were predicted as the active sites for PLD enzymatic activity, of which only H44 (H2O without a signal peptide) was confirmed [43]. Next, we performed molecular docking of SM to wild-type PLD and its aforementioned site-directed mutants using Autodock and Ligplot+. SM was found to be perfectly bound to the active pocket of PLD (Additional file 7), with a binding affinity of -5.8 kcal/mol (Additional file 8), indicating strong hydrogen bonding and hydrophobic interactions at the binding pocket [44]. Mutations of PLD at the above-predicted sites decreased its binding energy towards SM, especially for PLD (K114N), which had a binding affinity of only − 4.2 kcal/mol (Additional file 8). Furthermore, compared with XH02Δ*pld:pld* infection, XH02Δ*pld: mpld* infection significantly decreased IL-1β secretion and GSDMD cleavage in macrophages (Figures 8C and D). Compared with the uninfected macrophages, XH02Δ*pld:pld* but not XH02Δ*pld* and XH02Δ*pld:mpld* infection significantly decreased the mitochondrial membrane potential (Additional file 9). In addition, pro-Caspase-1 and GSDMD cleavage, IL-1β secretion, LDH release, and PI uptake were lower in pCMV-*pld* (K114N)-transfected macrophages than in pCMV-*pld*-transfected cells, and the disruption of the mitochondrial network structure was weaker in XH02Δ*pld:pld* (K114N)-infected macrophages than in XH02Δ*pld:pld*-infected cells (Additional file 9). Furthermore, the serum level of IL-1β in mice infected with XH02Δ*pld:mpld* was lower than that in XH02Δ*pld:pld*-infected mice (Figure 8E). In mice infected with XH02Δ*pld*:*mpld*, no mortality was observed within the 14-day observation period, whereas infection with XH02Δ*pld*:*pld* resulted in a mortality rate of 44.44% (Figure 8F). Abscesses were exclusively observed in the livers of mice infected with XH02 and XH02Δ*pld*:*pld* (Figure 8G). These results indicate that multiple enzyme active sites, including D66, G80, K114, and G242, are crucial for PLD-induced pyroptosis and are involved in the pathogenicity of Cp in mice.

### PLD targets mitochondria rather than cell membrane sphingomyelins and induces mitochondrial cardiolipin externalization to the outer mitochondrial membrane

**Figure 9:**
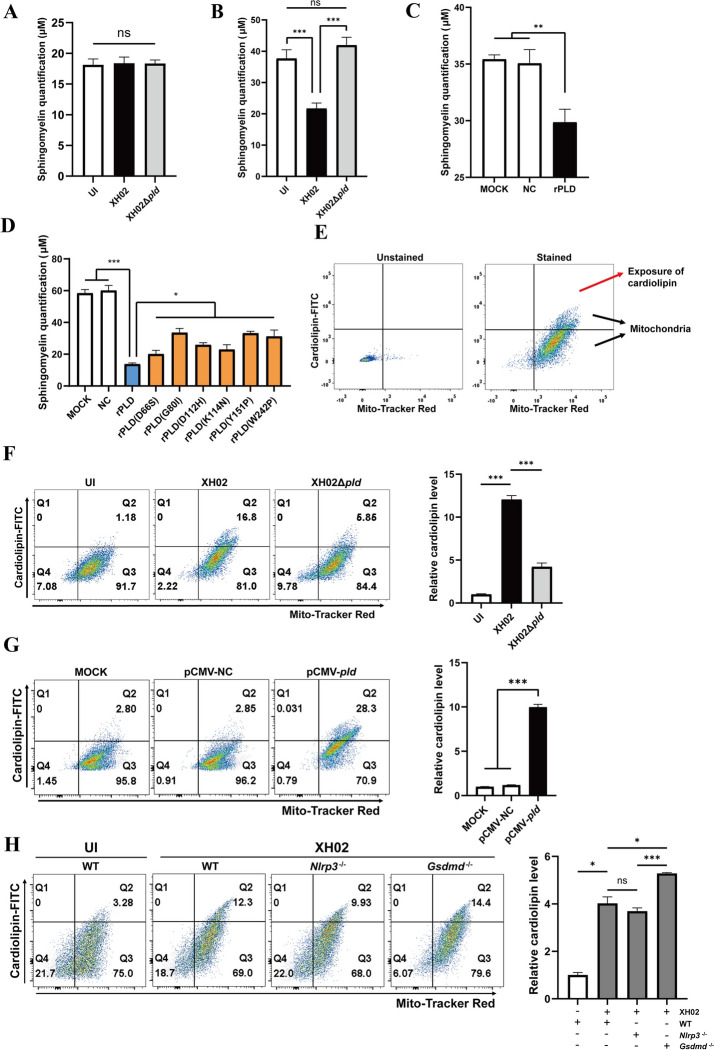
**PLD targets mitochondrial sphingomyelins and induces mitochondrial cardiolipin externalization to the outer mitochondrial membrane. A-B** J774A.1 cells were infected with XH02 (MOI = 10) for 12 h. The cell membranes and mitochondria were subsequently isolated. Sphingomyelin (SM) concentrations were measured in cell membranes (**A**) and mitochondria (**B**) (*n* = 9). **C** Mitochondria from PMs were isolated and incubated with rPLD (200 μg/mL) at 37 °C for 4 h, after which the SM concentration was determined (*n* = 9). **D** Mitochondria were isolated from PMs and incubated with rPLD (200 μg/mL) or mrPLD (200 μg/mL) at 37 °C for 4 h. The SM concentration was measured (*n* = 9). **E** Flow cytometry gating strategy for isolated mitochondria. The mitochondria were labelled with Annexin V and MitoTracker to measure the abundance of cardiolipin on the mitochondrial surface. **F** J774A.1 cells were infected with XH02 (MOI = 10) for 12 h, after which the externalization of cardiolipin was measured by flow cytometry (*n* = 9). **G** HEK293T cells were transfected with pCMV-*pld* for 12 h, and the externalization of cardiolipin was measured by flow cytometry (*n* = 9). **H** WT, *Nlrp*3^−/−^ and *Gsdmd*^−/−^ J774A.1 cells were infected with XH02 (MOI = 10) for 12 h, and the externalization of cardiolipin was measured by flow cytometry (*n* = 3). A to D, F, and G are pooled from three independent experiments. H is representative of three independent experiments. The error bars represent the SEMs. Statistical significance was determined by two-tailed Student’s *t* test: ***P < 0.001, **P < 0.01, *P < 0.05, ns ≥ 0.05.

The plasma membrane of mammalian cells is asymmetric in terms of phospholipid content, and sphingomyelins (SMs) are predominantly located in the outer layer [45, 46]. The target of PLD secreted by Cp within macrophages may not be the SMs on the plasma membrane [45]. The mitochondrial membrane is a bilayer membrane with phospholipids and cholesterol, and the SMs on the mitochondrial membrane may be a potential target for PLD. To verify this hypothesis, we detected the SMs on mitochondria and the plasma membrane from macrophages infected with Cp. No significant difference in the levels of plasma membrane SMs was detected among uninfected, XH02-infected or XH02Δ*pld-*infected macrophages (Figure 9A). However, compared with that in uninfected or XH02Δpld-infected cells, the level of SMs in mitochondria isolated from macrophages infected with XH02 was significantly lower (Figure 9B). In addition, the degradation of mitochondrial SMs by PLD was further confirmed by treating mitochondria with recombinant PLD (rPLD) (Figure 9C). Furthermore, the degradation capacity of rPLD for SM was significantly greater than that of mrPLD (Figure 9D). These data indicate that PLD secreted by Cp within macrophages targeted and degraded mitochondrial SMs but not cell membrane SMs.

Cardiolipin (CL) is the main type of phospholipid in mitochondria, and most CL molecules in mammalian cells are located in mitochondrial membranes, especially inner mitochondrial membranes (IMMs) [47, 48]. CL externalization to outer mitochondrial membranes (OMMs) implies mitochondrial damage, and CL on the OMM has been reported to bind with GSDMD-N and accelerate pyroptosis [28, 49, 50]. Annexin V binds to the CL of mitochondria [51], and Annexin V-FITC labelling effectively measures CL translocation to the OMM in the mitochondria [28, 29]. We analysed the CL externalization of mitochondria using flow cytometry after performing Annexin V-FITC and MitoTracker Red staining (Figure 9E) and reported that the CL externalization of mitochondria from XH02-infected PMs had an approximately threefold greater mean fluorescence intensity (MFI) than mitochondria from the XH02Δ*pld*-infected cells (Figure 9F). pCMV-*pld* transfection in J774A.1 cells also induced the externalization of CL (Figure 9G). These results confirmed that the PLD of Cp led to the externalization of CL to the OMM.

In addition, the absence of NLRP3 did not affect CL externalization in macrophages after Cp infection (Figure 9H). Furthermore, the transfection of pCMV-*pld* in HEK293T cells, which do not express NLRP3 inflammasome components [52], increased CL externalization (Additional file 10). However, GSDMD deficiency significantly promoted CL externalization during Cp infection (Figure 9H). Similar results were observed for the externalization of CL on mitochondria from Cp-infected macrophages treated with DSF (Additional file 10), an inhibitor of GSDMD [31]. These data indicate that the degree of CL externalization to the OMM induced by PLD in macrophages is not related to NLRP3 inflammasome levels but is negatively correlated with GSDMD levels.

## Discussion

Cp infection causes chronic inflammatory diseases characterized by a significant increase in the levels of inflammatory cytokines [4, 53]. We previously reported that the NLRP3 inflammasome plays a critical role in IL-1β secretion in Cp-infected macrophages [19] and that the deletion of *pld* decreases mortality and proinflammatory cytokine levels in the ascites and organs of Cp-infected mice [20], suggesting that the induction of inflammation promotes the pathogenesis of Cp infection. However, the precise mechanism remains elusive.

In the present study, we confirmed that Cp infection led to macrophage pyroptosis, which was characterized by LDH release, IL-1β secretion, and GSDMD cleavage in infected macrophages. Moreover, inhibition of pyroptosis by DSF and DMF, which are inhibitors that target GSDMD and the NLRP3 inflammasome [31, 32], prevented the release of Cp from infected macrophages to the culture medium and decreased the spread of Cp in macrophages. In addition, treatment of macrophages with antibodies against both Gly and NINJ1, which target NINJ1, also decreased the escape of Cp from infected macrophages and the spread of Cp in macrophages, confirming that pyroptosis facilitates the escape of Cp from infected macrophages and promotes the dissemination of this pathogen. These results are similar to the release of *M. tuberculosis* into the medium after THP-1 pyroptosis, which subsequently promotes bacterial dissemination [18], as well as the escape of *C. albicans* from infected macrophages after GSDMD-induced pyroptosis in [17]. Furthermore, treatment with DSF and DMF or *Nlrp3* knockout in mice effectively reduced the mortality caused by Cp infection, whereas LPS treatment accelerated the death of Cp*-*infected mice*,* suggesting that reducing the inflammatory response caused by pyroptosis in mice may be an effective way to reduce the death of Cp-infected mice.

Multiple signalling pathways, including canonical and noncanonical inflammasome pathways, mediate pyroptosis [11]. In canonical inflammasome pathways, the activation of the NLRP3 inflammasome generates active caspase-1, which cleaves GSDMD to GSDMD-N, thereby inducing pyroptosis [14]. This study confirmed that Cp infection resulted in macrophage pyroptosis, which is dependent on NLRP3 inflammasome activation, although *Nlrp3* knockout did not completely abolish GSDMD cleavage in Cp*-*infected or pCMV-*pld*-transfected macrophages. NLRP3 inflammasome activation generally requires two signals: signal 1 is the priming signal, which increases the expression of NLRP3 and pro-IL-1β, and signal 2 is the activation signal, which induces NLRP3 inflammasome assembly, caspase-1 activation, and pro-IL-1β and GSDMD cleavage [54]. The PLD of Cp is a sphingomyelin-specific phospholipase that catalyses the hydrolysis of sphingomyelin to ceramide phosphate and choline [7] and plays critical roles in the survival and dissemination of this pathogen within the host [8, 45, 55]. We found that deletion of *pld* did not affect the expression of NLRP3, pro-caspase-1, or pro-IL-1β but did reduce the levels of caspase-1 p20 and IL-1β in Cp-infected macrophages, and treatment of macrophages with culture supernatants from XH02 and XH02Δ*pld* did not obviously affect NLRP3 expression, suggesting that PLD is not involved in NLRP3 priming but is involved in NLRP3 inflammasome activation during infection by this pathogen.

Mitochondria are the energy production centres of cells and the driving forces through which the host responds to pathogenic infections and also play important roles in determining cell fate, including pyroptosis [56, 57]. Pathogens often target mitochondria and promote their infection by manipulating and damaging them to induce cell death [58, 59]. Mitochondrial damage leads to increased mtROS levels and cardiolipin externalization, which play key roles in triggering cell pyroptosis [49, 57]. XH02 or XH02Δ*pld:pld* infection, but not XH02Δ*pld* infection, increased mtROS levels and decreased mitochondrial membrane potential in macrophages, causing mitochondrial network structure disorganization, cristae disruption, and even mitochondria rupture, suggesting that PLD is involved in the process of macrophage mitochondrial dysfunction caused by Cp infection. Furthermore, inhibition of mtROS, mPTP, and ETC-generated ATP mitigated the activation of NLRP3 and pyroptosis in Cp-infected macrophages, prevented the escape of Cp from the culture supernatant and the spread of Cp to other macrophages, confirming the critical role of mitochondrial dysfunction in NLRP3 activation and, subsequently, pyroptosis in Cp*-*infected macrophages. Collectively, the results of this study revealed that PLD caused mitochondrial dysfunction and subsequent pyroptosis, promoting the escape and spread of Cp to other macrophages.

The PLD of Cp is a sphingomyelin-specific phospholipase that catalyses the hydrolysis of sphingomyelin to ceramide phosphate and choline [7]. We confirmed that the enzyme activity of PLD is associated with the induction of macrophage pyroptosis, as treatment with GW4869, a sphingomyelin hydrolysis inhibitor [42], reduced the pyroptosis of macrophages induced by PLD. We further identified D66, G80, K114, and G242 as key enzyme-active sites of PLD responsible for macrophage pyroptosis and Cp pathogenicity, since mutation of these sites reduced the binding affinity between PLD and SM; XH02Δ*pld:mpld* resulted in lower IL-1β secretion and GSDMD cleavage than XH02Δ*pld:pld* did in infected macrophages, and XH02Δ*pld:mpld* resulted in lower mortality than XH02Δ*pld:pld* did in infected mice.

The mitochondrial membrane is a bilayer membrane composed of phospholipids and cholesterol [47]. We found that Cp infection led to a significant decrease in the level of mitochondrial SMs but not plasma membrane SMs, that *pld* deletion abolished the decrease in mitochondrial SM level, that treatment with rPLD decreased the level of mitochondrial SMs, and that mutation of PLD decreased its ability to hydrolyse mitochondrial SMs, suggesting that the PLD of Cp within macrophages targets mitochondrial SMs but not plasma membrane SMs in macrophages.

Mitochondrial dysfunction is an early event in GSDMD-mediated pyroptosis [49]. CL is a target of NLRP3 and GSDMD-N binding and plays key roles in NLRP3 inflammasome activation [60], mitochondrial membrane permeabilization, and increased pyroptosis [49]. CL is predominantly located on the IMMs of mitochondria, and only small amounts of CL are present on the OMM under basal conditions [49]; the externalization of CL amplifies mitochondrial dysfunction and pyroptosis. We found that Cp infection caused the externalization of mitochondrial CL, and PLD plays a critical role in this process since *pld* deletion resulted in a significant decrease in mitochondrial CL externalization, whereas the transfection of pCMV-*pld* in J774A.1 cells induced the externalization of mitochondrial CL. Furthermore, we found that the externalization of the CL induced by PLD did not require the NLRP3 inflammasome pathway because PLD induced similar externalization of the CL in mitochondria from Cp*-*infected WT and *Nlrp3*^*−/−*^ macrophages. Interestingly, knockout of *Gsdmd*, or inhibition of GSDMD with DSF [31], caused significant increases in CL externalization of the mitochondria isolated from the Cp*-*infected macrophages, suggesting that GSDMD negatively regulates mitochondrial CL externalization induced by Cp infection; however, further research is needed.

In conclusion, our work revealed that Cp escapes from infected macrophages through PLD-induced pyroptosis. First, Cp infection upregulated NLRP3 expression in macrophages; then, the SMs of mitochondria were hydrolysed by PLD secreted from Cp within macrophages, resulting in mitochondrial dysfunction. Finally, CL externalization and mtROS release activated the NLRP3-GSDMD axis and subsequently induced macrophage pyroptosis, facilitating Cp escape and spread. Therefore, inhibiting the activation of the NLRP3 inflammasome and the occurrence of pyroptosis are among the strategies used to control Cp infection.

## Supplementary Information

**Additional file 1. Bacterial strains and plasmids used in this study.**

**Additional file 2. Primers used in this study.**

**Additional file 3. Effect of NLRP3 on the survival of Cp-infected mice.** **A** C57BL/6 mice were pretreated with DMF (50 mg/kg) by intraperitoneal injection at 24 and 4  h before intraperitoneal challenge with XH02 (6 × 105 CFU/mouse) (*n* = 5) and monitored for 14 days. **B** WT (*n* = 7) and Nlrp3^−/−^ (*n* = 5) C57BL/6 mice were intraperitoneally injected with XH02 (2 × 105 CFU/mouse) (*n* = 5) and monitored for 14 days.

**Additional file 4. Treatment with DSF, DMF, or Gly alleviates pyroptosis in Cp-infected macrophages.** **A** PMs were pretreated with DMF (20 μM) or DSF (10 μΜ) for 1 h and infected with XH02 (MOI = 10) for 12 h. The percentage of PI-positive PMs was determined by counting 3 randomly chosen visual fields (*n* = 3). **B** PMs were pretreated with DMF (20 μM), DSF (10 μΜ), or DMSO for 1 h and infected with XH02 (MOI = 10) for 12 h, after which the IL-1β levels in the supernatants were measured (*n* = 3). **C** Expression of GSDMD in PMs pretreated with DMF (20 μM) or DSF (10 μΜ) for 1 h and infected with XH02 (MOI = 10) for 24 h. **D** J774A.1 cells were infected with XH02 (MOI = 10) and treated with Gly (10 mM) for 12 h, after which LDH release was determined (*n* = 3). A, B, and D are representative of three independent experiments. The error bars represent the SEMs. Statistical significance was determined by two-tailed Student’s *t* test: **P < 0.01, *P < 0.05.

**Additional file 5. Transcriptome sequencing analysis of C. pseudotuberculosis-infected PMs and quantitative real-time PCR validation**. **A** Heatmap of differentially expressed genes from the transcriptome sequencing of PMs infected with XH02 or XH02Δ*pld* (MOI = 10) for 4 h. RNA-seq was performed with three independent biological replicates per group (*n* = 3). **B** Heatmap showing the expression of 18 genes, including pyroptosis-associated genes, in PMs infected with XH02 or XH02Δpld (MOI = 10); colour intensity represents normalized gene expression (z score). The data represent three independent samples. **C** qPCR validation of genes from transcriptome sequencing of PMs infected with XH02 or XH02Δ*pld* (MOI = 10) (*n* = 6). The error bars represent the SEMs. Statistical significance was determined by two-tailed Student’s *t* test.

**Additional file 6. PLD is involved in NINJ1 oligomerization in Cp-infected macrophages.** PMs were infected with *sfGFP*-labelled (green) XH02 or XH02Δ*pld* (MOI = 10) for 12 h, followed by immunofluorescence staining with anti-NINJ1 (red). The nuclei were stained with DAPI (blue), and images were captured by laser confocal microscopy. The greyscale images show merged channels, with red arrows indicating NINJ1 oligomerization.

**Additional file 7. Molecular docking of PLD and sphingomyelin (SM).** **A** Docking diagram illustrating the interaction between PLD and SM. **B** Docking analysis of PLD or mrPLD with SM performed using LigPlot+.

**Additional file 8. Autodock molecular docking results of PLD or mutant PLD to MS.**

**Additional file 9. Site mutation of PLD affects the mitochondrial membrane potential and pyroptosis of macrophages.** **A** J774A.1 cells were infected with XH02Δ*pld*, XH02Δ*pld:pld*, or XH02Δ*pld:mpld* (MOI = 10) for 4 h. The mitochondrial membrane potential was analysed using a JC-1 kit (*n* = 4). **B‒C** J774A.1 cells were transfected with pCMV-*pld* or pCMV-*pld (K114N)* for 12 h. The expression of NLRP3, GSDMD and Caspase-1 and the percentages of PI-positive cells, LDH release and IL-1β secretion were measured (*n* = 3). D PMs were infected with XH02Δ*pld:pld* and XH02Δ*pld:pld(K114N)* (MOI = 10) for 12 h. Images of anti-TOM20 antibodies (green) and DAPI (blue)-stained macrophages were taken by laser confocal microscopy. **A** and **C** are representative of three independent experiments. The error bars represent the SEMs. Statistical significance was determined by two-tailed Student’s *t* test: ***P < 0.001, *P < 0.05.

**Additional file 10. PLD induces the externalization of cardiolipin.** **A** J774A.1 cells were transfected with pCMV-*pld* for 12 h. Cardiolipin externalization of mitochondria was measured by flow cytometry (*n* = 9). **B** J774A.1 cells were pretreated with DSF (10 μM) for 1 h and infected with XH02 (MOI = 10) for 12 h. Cardiolipin externalization of mitochondria was measured by flow cytometry (*n* = 6). A is pooled from three independent experiments. B is pooled from two independent experiments. The error bars represent the SEMs. Statistical significance was determined by two-tailed Student’s *t* test: ***P < 0.001.

## Data Availability

All the data generated or analysed during this study are included in this published article and its supplementary information files.
