# Supplementary material for: Corynebacterium pseudotuberculosis phospholipase D targets mitochondrial sphingomyelin and induces NLRP3-GSDMD axis-mediated pyroptosis in macrophages to promote infection
Source: Vet Res. 2025 Oct 16;56:198. doi: 10.1186/s13567-025-01640-7 (PMC12533471; doi:10.1186/s13567-025-01640-7)
Supplement: Supplementary file 1 — Additional file 1. Bacterial strains and plasmids used in this study. [file 13567_2025_1640_MOESM1_ESM.docx]

**Additional file 1 Bacterial strains and plasmids used in this study.**

| **Strains or plasmids** | **Description^a^** | **Source** |
| --- | --- | --- |
| **Strains** |  |  |
| DH5α | *E. coli*, cloning strain | TransGen Biotech |
| BL21 (DE3) | *E. coli*, expressing strain | TransGen Biotech |
| XH02 | *C. pseudotuberculosis* clinical isolated strain. | Lab stored |
| XH02Δ*pld* | Derived from XH02, *pld* deleted strain. | Lab stored |
| XH02-*sfGFP* | XH02 with pXMJ19-*sfGFP* that constitutively express sfGFP.. | This study |
| XH02Δ*pld*-*sfGFP* | XH02Δ*pld* with XH02 with pXMJ19-*sfGFP* that constitutively express sfGFP.. | This study |
| XH02Δ*pld:pld* | Complementary expression of PLD in XH02Δ*pld*, Cm^R^ | This study |
| XH02Δ*pld:pld*(D66S) | Complementary expression of mutant PLD (D66S) in XH02Δ*pld*, Cm^R^ | This study |
| XH02Δ*pld:pld*(G80I) | Complementary expression of mutant PLD (G80I) in XH02Δ*pld*, Cm^R^ | This study |
| XH02Δ*pld:pld*(K114N) | Complementary expression of mutant PLD (K114N) in XH02Δ*pld*, Cm^R^ | This study |
| XH02Δ*pld:pld*(W242P) | Complementary expression of mutant PLD (W242P) in XH02Δ*pld*, Cm^R^ | This study |
| XH02Δ*pld:mpld* | Complementary expression of mutant PLD at D66S, G80I, K114N, or W242P in XH02Δ*pld* | This study |
| **Plasmids** |  |  |
| pXMJ19 | *E. coli*-*C. glutamicum* shuttle vector. | Provided by Dr. Jibing Sun |
| pXMJ19-*sfGFP* | Removed *lacIq* from pXMJ19, constitutively expression of sfGFP, Cm^R^ | Lab store |
| pXMJ19-*pld* | Derived from pXMJ19*-sfGFP*, constitutively expression of PLD, Cm^R^ | This study |
| pCMV-NC | pCMV negative control | Lab stored |
| pCMV-*pld* | pCMV plasmid expressing PLD | This study |
| pCMV-*pld*(K114N) | pCMV plasmid expressing PLD (K114N) | This study |
| pCold-NC | pCold-TF, negative control | Lab stored |
| pCold-*pld* | Derived from pCold-TF, expression of PLD | This study |
| pCold-*pld*(D66S) | Derived from pCold-TF, expression of PLD (D66S), Amp^R^ | This study |
| pCold-*pld*(G80I) | Derived from pCold-TF, expression of PLD (G80I), Amp^R^ | This study |
| pCold-*pld*(D112H) | Derived from pCold-TF, expression of PLD (D112H), Amp^R^ | This study |
| pCold-*pld*(K114N) | Derived from pCold-TF, expression of PLD (K114N), Amp^R^ | This study |
| pCold-*pld*(Y151P) | Derived from pCold-TF, expression of PLD (Y151P), Amp^R^ | This study |
| pCold-*pld*(W242P) | Derived from pCold-TF, expression of PLD (W242P), Amp^R^ | This study |
| LentiCRISPRv2-mCherry | Lentiviral vector encoding sgRNA cloning site + hSpCAS9-P2A-mCherry, Amp^R^ | Addgene |
| psPAX2 | 2^nd^ generation lentiviral packaging plasmid, Amp^R^ | Addgene |
| pMD2.G | VSV-G envelope expressing plasmid, Amp^R^ | Addgene |

^a^**Km^R^, Cm^R^, and Amp^R^ represent resistance to kanamycin, chloramphenicol, and ampicillin respectively.**
