# Supplementary material for: Corynebacterium pseudotuberculosis phospholipase D targets mitochondrial sphingomyelin and induces NLRP3-GSDMD axis-mediated pyroptosis in macrophages to promote infection
Source: Vet Res. 2025 Oct 16;56:198. doi: 10.1186/s13567-025-01640-7 (PMC12533471; doi:10.1186/s13567-025-01640-7)
Supplement: Supplementary file 2 — Additional file 2. Primers used in this study. [file 13567_2025_1640_MOESM2_ESM.docx]

**Additional file 2 Primers used in this study**

| **Primer name** | **Sequence (5’-3’)** | **Purpose** |
| --- | --- | --- |
| SD-sfGFP-F | CCCAAGCTTGAAAGGAGGTTTATGAGCAAAGGAGAAGAACTTTTCA | Amplification of *sfGFP* fragment |
| SD-sfGFP-R | CCGGAATTCTTATTTGTAGAGCTCATCCATGCCA |  |
| pXMJ19-JD-F | TTGACAATTAATCATCGGCTCGT | Identification of pXMJ19 plasmid |
| pXMJ19-JD-R | CTCTCATCCGCCAAAACAGCC |  |
| pX-sdpld-F（Hind Ⅲ） | CCCAAGCTTGAAAGGAGGTTTATGAGGGAGAAAGTTGTTTTATTC | Amplification of *pld* fragment |
| pX-sdpld-R（EcoRⅠ） | CCGGAATTCTCAATGATGATGATGATGATGCCACGGGTTATCCGCTAC |  |
| PLD-D66S-F | CGTTAGAAATTAGCTTCACTGCGTGGGGTCGT | Construction of mutant PLD at 66^th^ amino acid |
| PLD-D66S-R | AGTGAAGCTAATTTCTAACGCATTCGCACCGAT |  |
| PLD-G80I-F | ATTATTCCTACTAGCGCAGGTGCTACTGCAGAGGAAA | Construction of mutant PLD at 80^th^ amino acid |
| PLD-G80I-R | ACCTGCGCTAGTAGGAATAATATCATGATCTGCC |  |
| PLD-D112H-F | CACATCAAGAATCCAGACTACTGCAGGGATGC | Construction of mutant PLD at 112^th^ amino acid |
| PLD-D112H-R | GTCTGGATTCTTGATGTGAAGCCAGGTGAA |  |
| PLD-K114N-F | GACATCAATAATCCAGACTACTGCAGGGATGCTCGTAGTG | Construction of mutant PLD at 114^th^ amino acid |
| PLD-K114N-R | GCAGTAGTCTGGATTATTGATGTCAAGCCAGGTGA |  |
| PLD-Y151P-F | ATGGGTTCCCTAAGACAGTCGGCGGACCTGC | Construction of mutant PLD at 151^th^ amino acid |
| PLD-Y151P-R | ACTGTCTTAGGGAACCCATAGAGAACTCGAACC |  |
| PLD-W242P-F | TTGGGCCGACAATCGCTACAGGTCAGGACGC | Construction of mutant PLD at 242^th^ amino acid |
| PLD-W242P-R | CCTGTAGCGATTGTCGGCCCAAAAGTTTTACCG |  |
| pCMVJD-F | AGGTCTATATAAGCAGAGCTGGTT | Identification of pCMV Plasmid |
| pCMVJD-R | CGAGTGAATTGTAATACGACTCAC |  |
| PLD-F(EcoRⅠ） | CCCGAATTCATGGCGCCTGTTGTGCATAACCC | Amplify the *pld* fragment for constructing pCMV-*pld* |
| PLD-FLAG-R | TGGTCTTTGTAGTCAAGCTTCCACGGGTTATCCGCTA |  |
| PLD-FLAG2-R | GTCGATGTCATGATCTTTATAATCACCGTCATGGTCTTTGTAGTCAAG |  |
| PLD-FLAG3-R(Xho Ⅰ) | CCGCTCGAGCTACTTGTCATCGTCATCCTTGTAGTCGATGTCATGATCT |  |
| pld-F | GTAAGTCCAGCGAAGCTCGT | qPCR primers for the *pld* |
| pld-R | ATCATTAACTCGCGCGTCCT |  |
| 16 S rRNA-F | CAGCTCGTGTCGTGAGATGT | qPCR primers for the *16S rRNA* |
| 16 S rRNA-R | CTCTCATGAGTCCCCACCAT |  |
| Gsdmd-sgRNA-F | TGCAACAGCTTCGGAGTCGT | Guide the CRISPR-Cas9 system to precisely target and cleave the *Gsdmd* sequence. |
| Gsdmd-sgRNA-R | ACGACTCCGAAGCTGTTGCA |  |
| Nlrp3-sgRNA-F | GTGTTGTCAGGATCTCGCAT | Guide the CRISPR-Cas9 system to precisely target and cleave the *Nlrp3* sequence. |
| Nlrp3-sgRNA-R | ATGCGAGATCCTGACAACAC |  |
