# Supplementary figures and images for: Corynebacterium pseudotuberculosis phospholipase D targets mitochondrial sphingomyelin and induces NLRP3-GSDMD axis-mediated pyroptosis in macrophages to promote infection
Source: Vet Res. 2025 Oct 16;56:198. doi: 10.1186/s13567-025-01640-7 (PMC12533471; doi:10.1186/s13567-025-01640-7)

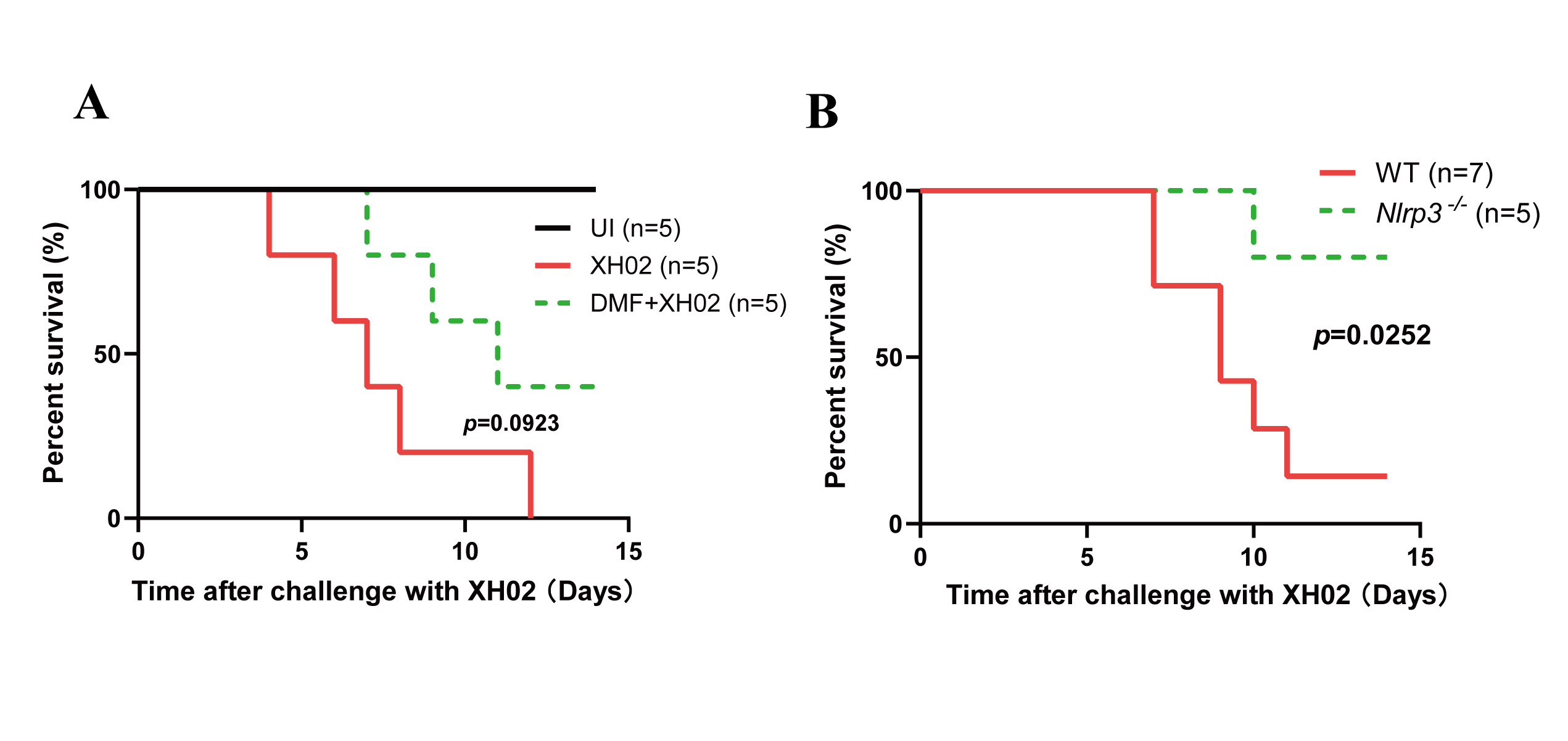

Supplement: Supplementary file 3 — Additional file 3. Effect of NLRP3 on the survival of Cp-infected mice. A C57BL/6 mice were pretreated with DMF (50 mg/kg) by intraperitoneal injection at 24 and 4 h before intraperitoneal challenge with XH02 (6 × 10^5^ CFU/mouse) (n = 5) and monitored for 14 days. B WT (n = 7) and Nlrp3−/− (n = 5) C57BL/6 mice were intraperitoneally injected with XH02 (2 × 10^5^ CFU/mouse) (n = 5) and monitored for 14 days. [file 13567_2025_1640_MOESM3_ESM.tif]

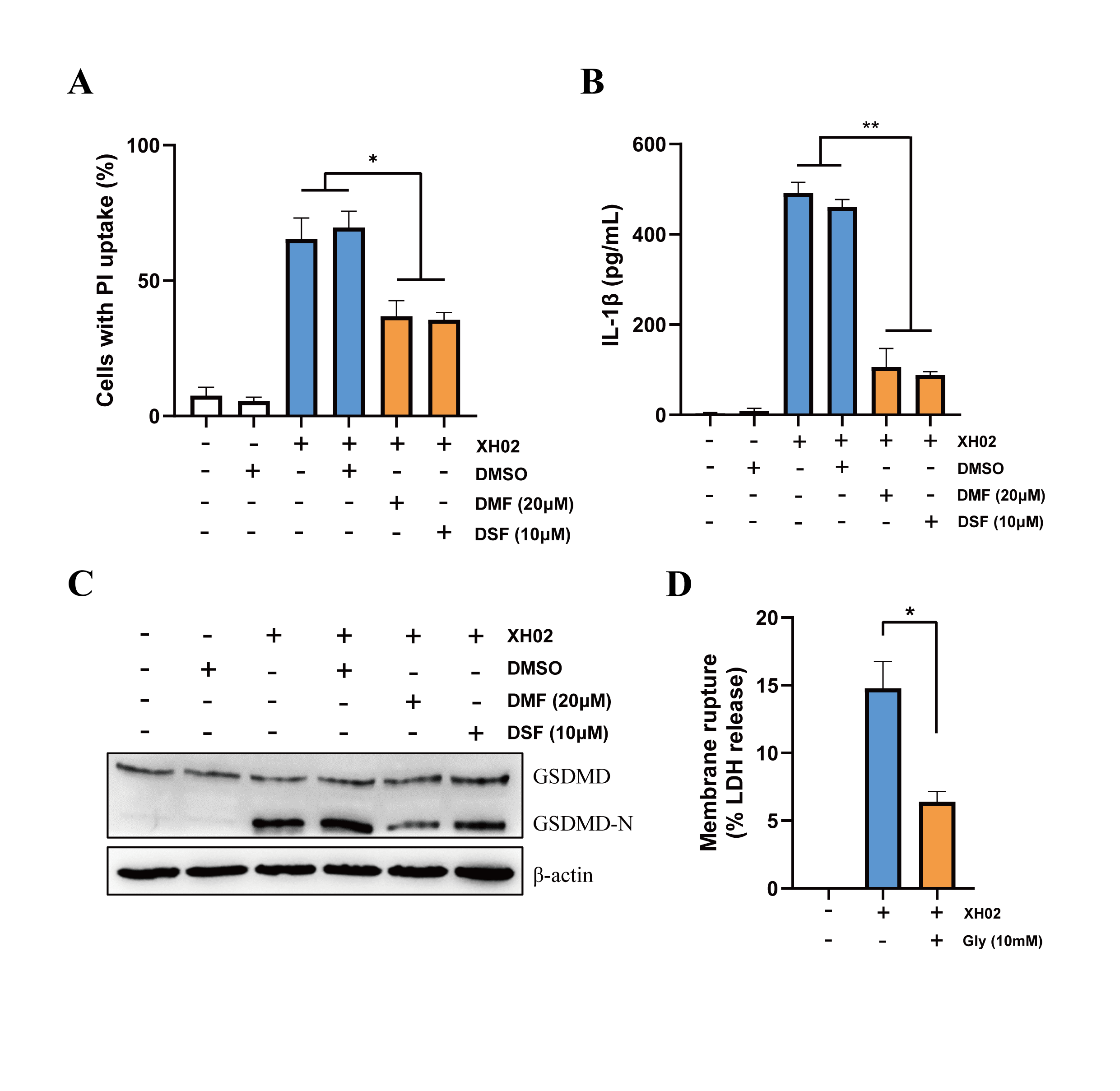

Supplement: Supplementary file 4 — Additional file 4. Treatment with DSF, DMF, or Gly alleviates pyroptosis in Cp-infected macrophages. A PMs were pretreated with DMF (20 μM) or DSF (10 μΜ) for 1 h and infected with XH02 (MOI = 10) for 12 h. The percentage of PI-positive PMs was determined by counting 3 randomly chosen visual fields (n = 3). B PMs were pretreated with DMF (20 μM), DSF (10 μΜ), or DMSO for 1 h and infected with XH02 (MOI = 10) for 12 h, after which the IL-1β levels in the supernatants were measured (n = 3). C Expression of GSDMD in PMs pretreated with DMF (20 μM) or DSF (10 μΜ) for 1 h and infected with XH02 (MOI = 10) for 24 h. D J774A.1 cells were infected with XH02 (MOI = 10) and treated with Gly (10 mM) for 12 h, after which LDH release was determined (n = 3). A, B, and D are representative of three independent experiments. The error bars represent the SEMs. Statistical significance was determined by two-tailed Student’s t test: **P < 0.01, *P < 0.05. [file 13567_2025_1640_MOESM4_ESM.tif]

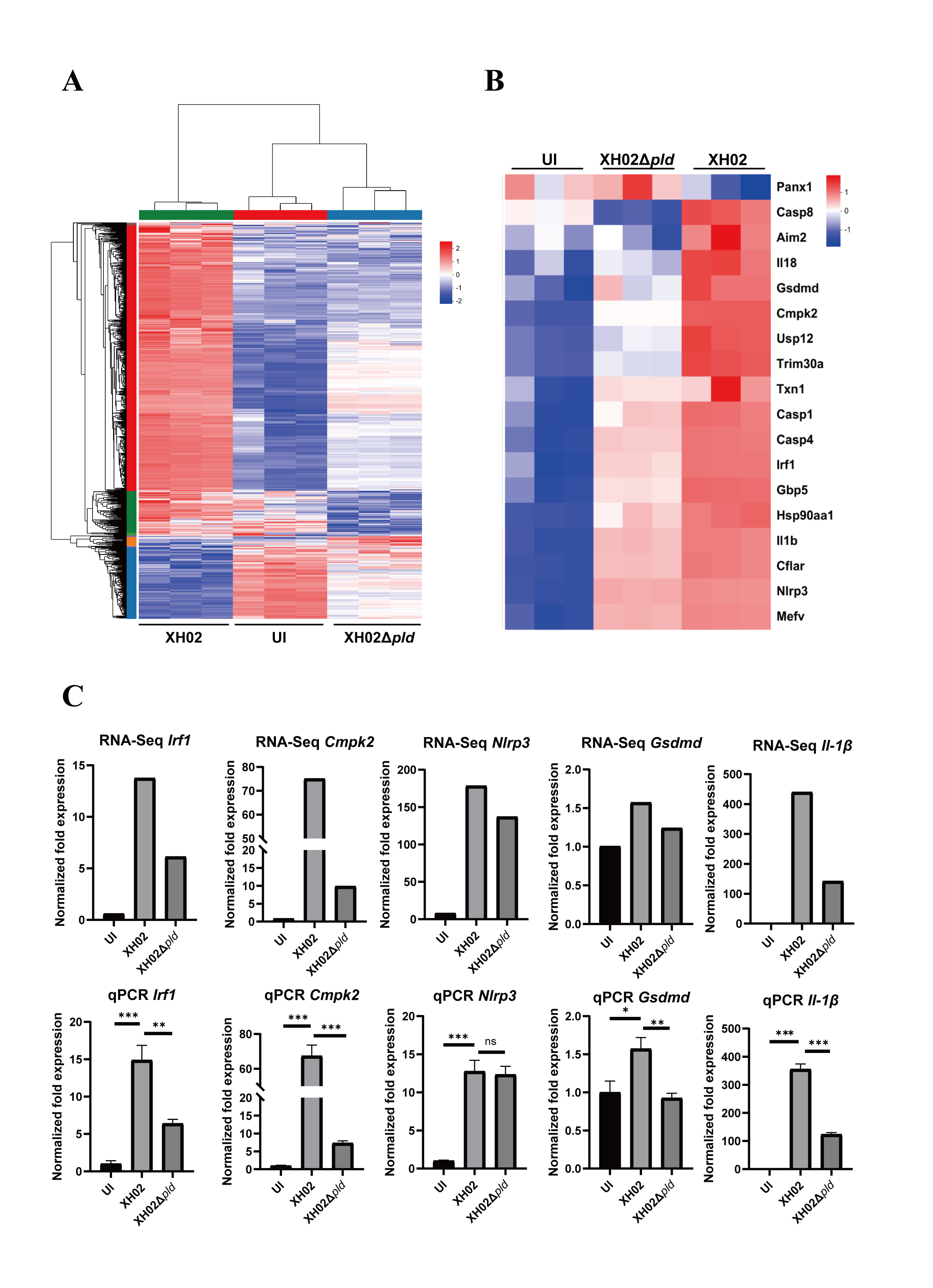

Supplement: Supplementary file 5 — Additional file 5. Transcriptome sequencing analysis of C. pseudotuberculosis-infected PMs and quantitative real-time PCR validation. A Heatmap of differentially expressed genes from the transcriptome sequencing of PMs infected with XH02 or XH02Δpld (MOI = 10) for 4 h. RNA-seq was performed with three independent biological replicates per group (n = 3). B Heatmap showing the expression of 18 genes, including pyroptosis-associated genes, in PMs infected with XH02 or XH02Δpld (MOI = 10); colour intensity represents normalized gene expression (z score). The data represent three independent samples. C qPCR validation of genes from transcriptome sequencing of PMs infected with XH02 or XH02Δpld (MOI = 10) (n = 6). The error bars represent the SEMs. Statistical significance was determined by two-tailed Student’s t test. [file 13567_2025_1640_MOESM5_ESM.tif]

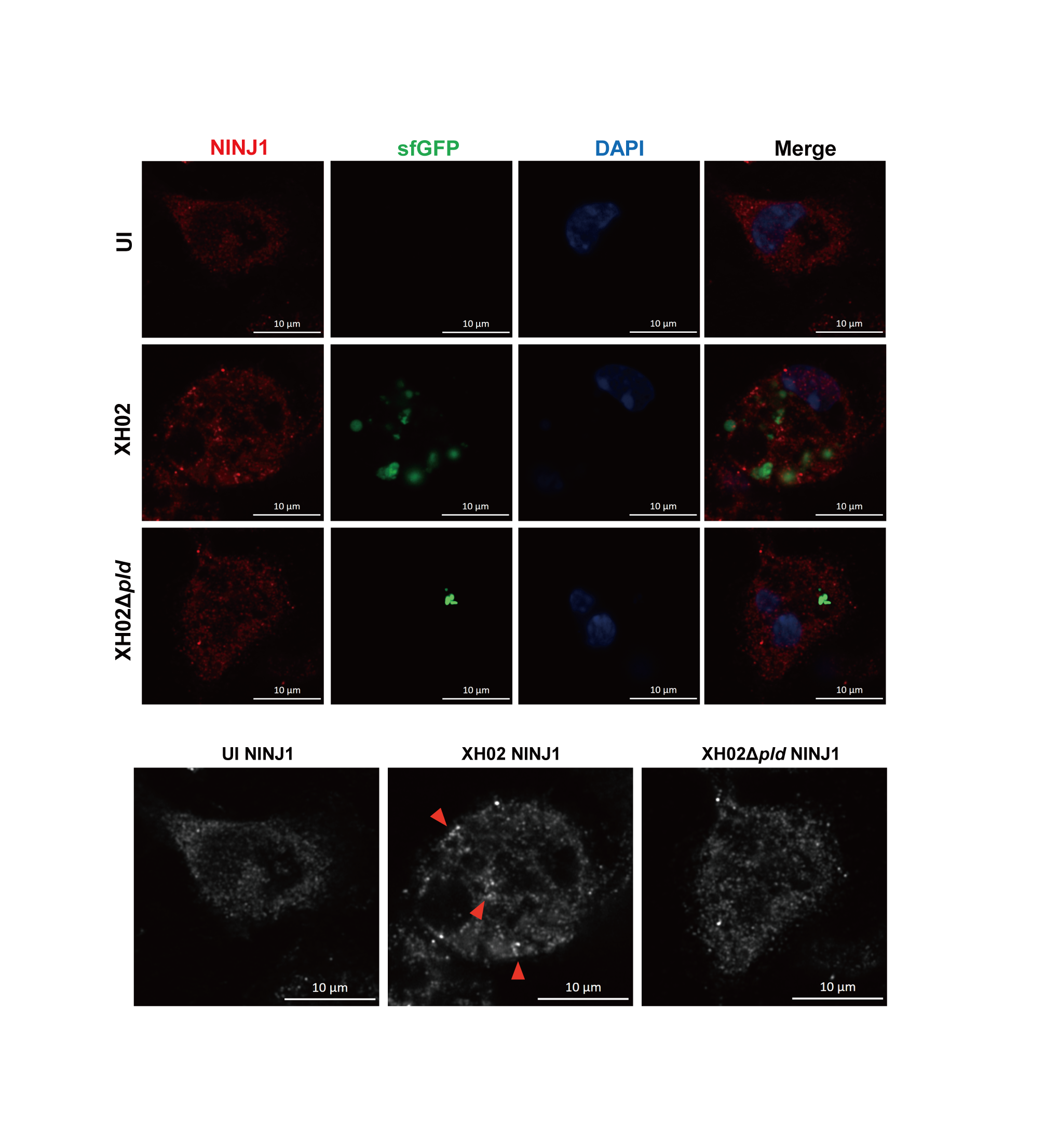

Supplement: Supplementary file 6 — Additional file 6. PLD is involved in NINJ1 oligomerization in Cp-infected macrophages. PMs were infected with sfGFP-labelled (green) XH02 or XH02Δpld (MOI = 10) for 12 h, followed by immunofluorescence staining with anti-NINJ1 (red). The nuclei were stained with DAPI (blue), and images were captured by laser confocal microscopy. The greyscale images show merged channels, with red arrows indicating NINJ1 oligomerization. [file 13567_2025_1640_MOESM6_ESM.tif]

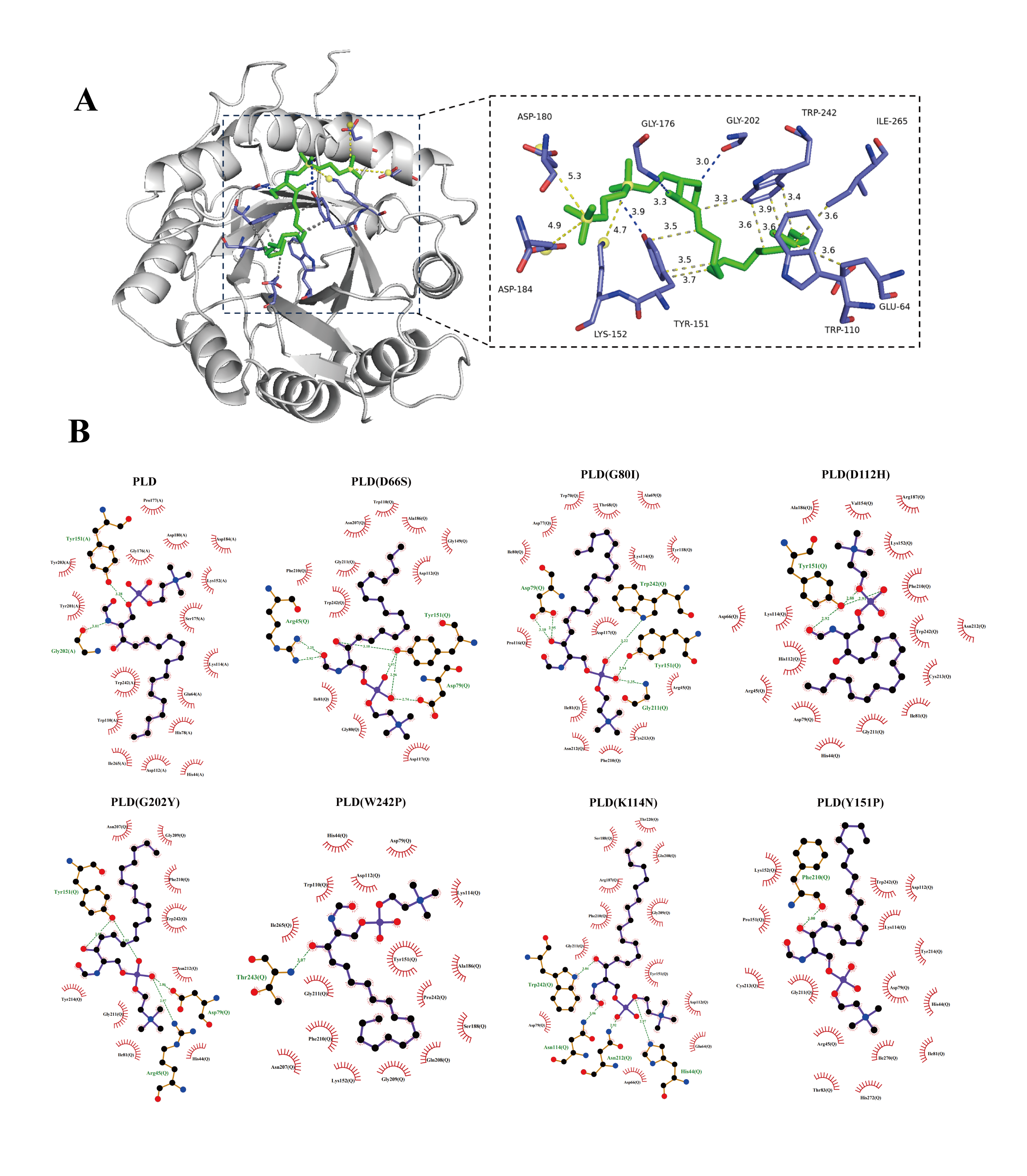

Supplement: Supplementary file 7 — Additional file 7. Molecular docking of PLD and sphingomyelin (SM). A Docking diagram illustrating the interaction between PLD and SM. B Docking analysis of PLD or mrPLD with SM performed using LigPlot+. [file 13567_2025_1640_MOESM7_ESM.tif]

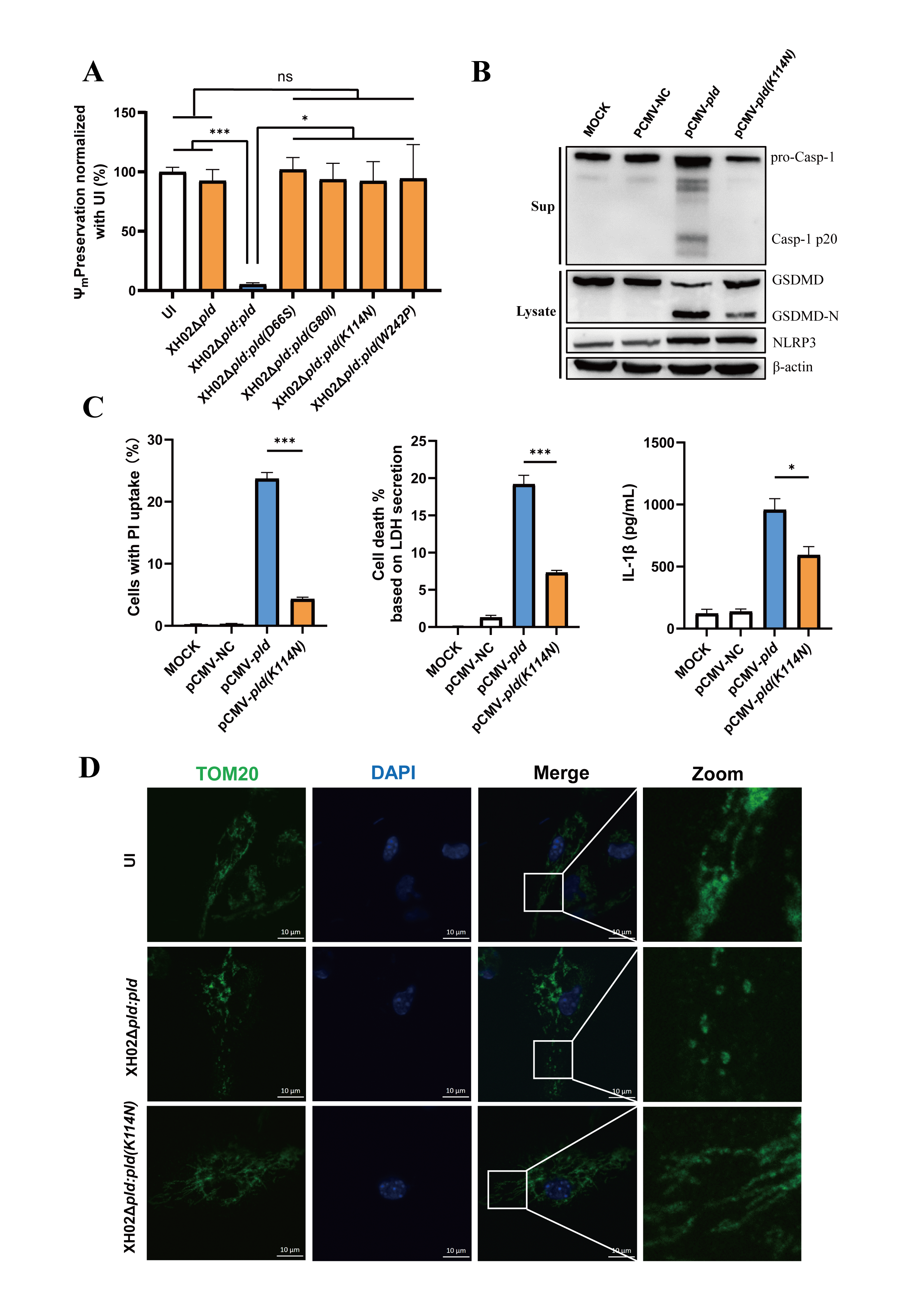

Supplement: Supplementary file 9 — Additional file 9. Site mutation of PLD affects the mitochondrial membrane potential and pyroptosis of macrophages. A J774A.1 cells were infected with XH02Δpld, XH02Δpld:pld, or XH02Δpld:mpld (MOI = 10) for 4 h. The mitochondrial membrane potential was analysed using a JC-1 kit (n = 4). B‒C J774A.1 cells were transfected with pCMV-pld or pCMV-pld (K114N) for 12 h. The expression of NLRP3, GSDMD and Caspase-1 and the percentages of PI-positive cells, LDH release and IL-1β secretion were measured (n = 3). D PMs were infected with XH02Δpld:pld and XH02Δpld:pld(K114N) (MOI = 10) for 12 h. Images of anti-TOM20 antibodies (green) and DAPI (blue)-stained macrophages were taken by laser confocal microscopy. A and C are representative of three independent experiments. The error bars represent the SEMs. Statistical significance was determined by two-tailed Student’s t test: ***P < 0.001, *P < 0.05. [file 13567_2025_1640_MOESM9_ESM.tif]

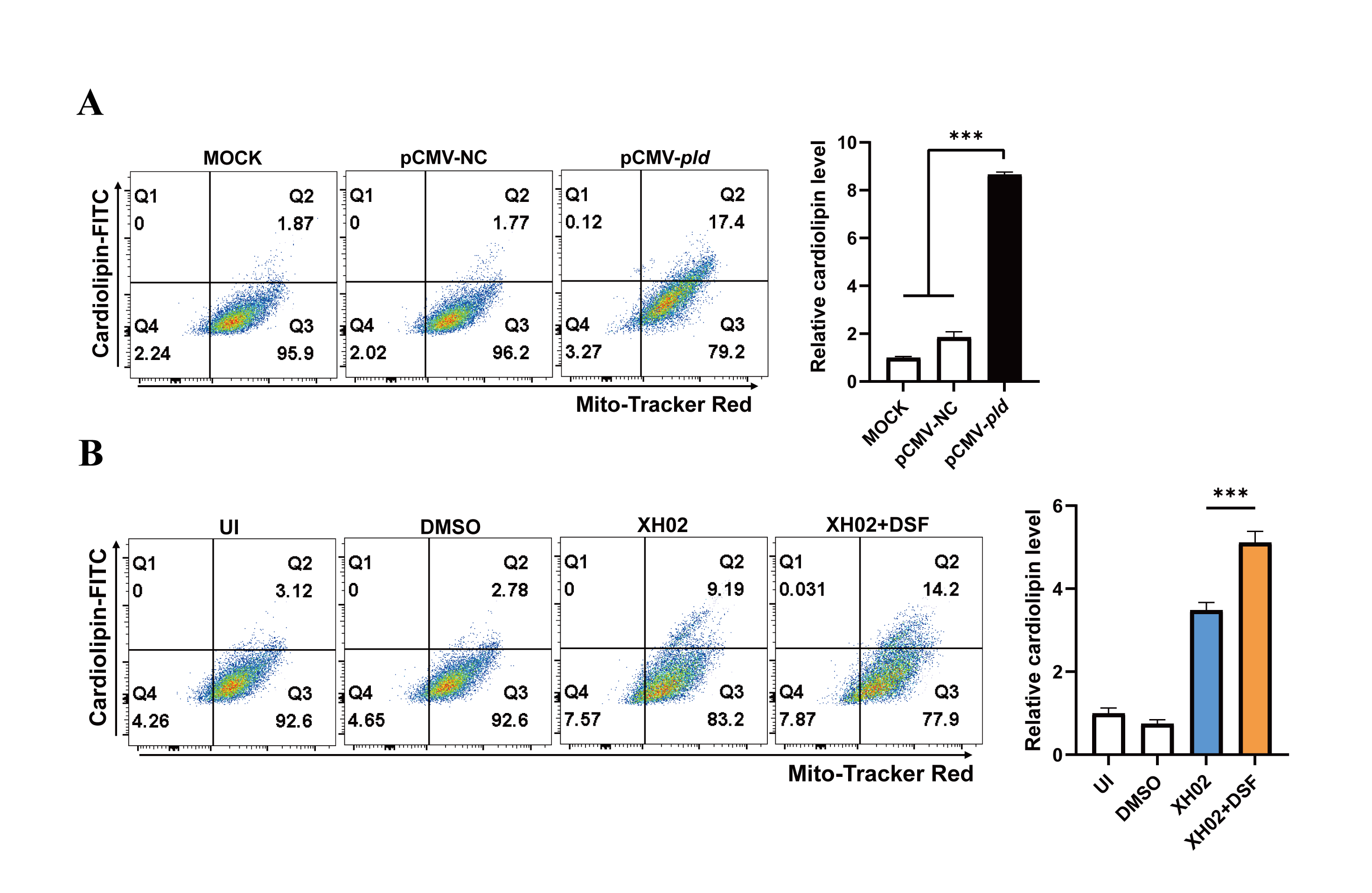

Supplement: Supplementary file 10 — Additional file 10. PLD induces the externalization of cardiolipin. A J774A.1 cells were transfected with pCMV-pld for 12 h. Cardiolipin externalization of mitochondria was measured by flow cytometry (n = 9). B J774A.1 cells were pretreated with DSF (10 μM) for 1 h and infected with XH02 (MOI = 10) for 12 h. Cardiolipin externalization of mitochondria was measured by flow cytometry (n = 6). A is pooled from three independent experiments. B is pooled from two independent experiments. The error bars represent the SEMs. Statistical significance was determined by two-tailed Student’s t test: ***P < 0.001. [file 13567_2025_1640_MOESM10_ESM.tif]
