# Supplementary material for: Corynebacterium pseudotuberculosis phospholipase D targets mitochondrial sphingomyelin and induces NLRP3-GSDMD axis-mediated pyroptosis in macrophages to promote infection
Source: Vet Res. 2025 Oct 16;56:198. doi: 10.1186/s13567-025-01640-7 (PMC12533471; doi:10.1186/s13567-025-01640-7)
Supplement: Supplementary file 8 — Additional file 8. Autodock molecular docking results of PLD or mutant PLD to MS. [file 13567_2025_1640_MOESM8_ESM.docx]

**Additional file 8 Autodock molecular docking result of PLD or mutant PLD to MS.**

| **Targets** | **Hydrophobic Interactions** | **Hydrogen Bonds** | **Salt Bridges** | **Energry**  **(Kcal/mol)** |
| --- | --- | --- | --- | --- |
| PLD | GLU64 TRP110 LYS152 ILE265 | TYR151 GLY176 GLY202 | LYS152 ASP180 ASP184 | -5.8 |
| PLD (D66S) | ASP112 ALA186 PHE210 TRP242 | ARG45 TYR151 | ASP117 | -5.7 |
| PLD (G80I) | THR68 TRP70 ILE80 LYS114 PRO116 | GLY211 TRP242 | ARG45 | -5.4 |
| PLD (D112H) | ARG45 ASP79 ILE81 PHE210 TRP242 | LYS114 TYR151 | None | -5.1 |
| PLD (K114N) | TYR151 GLN208 THR220 | ARG45 ASP79 ASN114 ASN212 TRP242 | HIS44 ASP66 ASP112 | -4.2 |
| PLD (Y151P) | LYS114 PRO151 PHE210 | GLY211 TRP242 | HIS44 ARG45 | -4.7 |
| PLD (G202Y) | PHE210 TRP242 | TYR151 | ARG45 | -5.1 |
| PLD (W242P) | TYR151 LYS152 ALA186 ASN207 | GLU64 PHE210 GLY211 ASN212 | ARG45 | -5.3 |
